# Efficient expression of a novel *α*-amylase for reduction of tobacco starch and smoke hazard

**DOI:** 10.3389/fmicb.2025.1603337

**Published:** 2025-07-09

**Authors:** Zongchen Han, Jie Hao, Dian Zou, Zhikang Sun, Zekun Zhang, Chenqi Niu, Qi Lu, Kuo Huang, Changwen Ye, Xuetuan Wei

**Affiliations:** ^1^Zhengzhou Tobacco Research Institute of China National Tobacco Corporation, Zhengzhou, China; ^2^Inner Mongolia Kunming Cigarettes LLC, Hohhot, China; ^3^State Key Laboratory of Agricultural Microbiology, Huazhong Agricultural University, Wuhan, China; ^4^Institute of Agro-Products Processing and Nuclear Agricultural Technology, Hubei Academy of Agricultural Sciences, Wuhan, China

**Keywords:** tobacco starch, *α*-amylase, *Bacillus amyloliquefaciens*, expression optimization, application evaluation

## Abstract

The combustion of excessive starch in tobacco leaves leads to more harmful substances, adversely affecting the sensory properties of tobacco and posing significant risks to human health. Therefore, there is an urgent need to develop specific amylases targeting tobacco starch to address these issues. In this study, 5 different *α*-amylase genes were selected for recombinant expression in *Bacillus amyloliquefaciens* BAX-5, and the *amyA(LC)* (derived from *Bacillus amyloliquefaciens* MK10163) was confirmed to be the optimal gene. Then, the *α*-amylase activity was further increased by screening host bacteria BAX-5 and signal peptides SP_003_ (derived from the *dacB* gene of *Bacillus subtilis* 168). Subsequently, the *α*-amylase properties were characterized, such as temperature tolerance, pH tolerance and metal ion. Through replacement of culture medium, the recombinant strain BAX-5/PT-17SP_003_*amyA(LC)* produced the maximum *α*-amylase activity of 904.91 IU/mL, which was about 4 times higher than that of the original culture medium. Finally, the *α*-amylase Amy (LC) was applied to the enzyme treatment of tobacco leaves, and the evaluation results showed that *α*-amylase Amy (LC) could play a positive role in reducing damage and enhancing quality of cigarettes. This research provides a novel enzymatic resource for the development of amylases, and it has enormous market potential and application value.

## 1 Introduction

Starch is one kind of abundant biological macromolecules in tobacco leaves, and the content of starch in flue-cured tobacco leaves can reach 40% (Chu et al., 2022). During the roasting of tobacco leaves, the high content of macromolecular substances (e.g., starch) often leads to incomplete combustion, releasing harmful compounds such as aromatic hydrocarbons and aldehydes, which are known to pose significant health risks (Ye et al., 2024; Tian et al., 2023). On the other hand, the high content of macromolecules can reduce the smoke properties and sensory qualities of tobacco, and the starch content have negative correlation with its total sensory evaluation score (Chen et al., 2017), which significantly affect quality of tobacco leaves (Yan et al., 2022). Therefore, reduction of the starch content in the tobacco has become a key issue to reduce the hazard and improve the quality (Yang et al., 2010).

There are many factors that can affect the starch content of tobacco leaf in the growth and cultivation stage. Several methods have been developed to reduce the starch content of tobacco (Yan et al., 2020). Through increase of baking time, the degradation degree of starch changes greatly (Sun et al., 2011). In recent years, application of microbial enzymes to degrade macromolecules has also been explored to improve tobacco quality and reduce the hazards, and the use of *α*-amylase to treat tobacco leaves has become the focus of the tobacco industry (Xing et al., 2023). However, present *α*-amylase used in tobacco are from commercial food enzymes, which are aimed to hydrolyze the starch in traditional cereals, and there are no enzymes specifically designed to degrade starch in tobacco leaves. Therefore, it is important to screen and optimize the amylases that target to degrade the starch in tobacco.

At present, *Bacillus* has become a general industrial microbial chassis cell, and nearly 60% of the enzyme products on the market are derived from *Bacillus* (Liu et al., 2023). *Bacillus* expression systems have strong protein secretion ability and high expression efficiency, which can rapidly secrete protease, amylase and lipase, and they also have good compatibility with foreign proteins (Cai et al., 2022). With the development of genetic engineering and molecular biology, *Bacillus* has been developed as the main expression system for recombinant proteins (Su et al., 2020; Mukherjee and Venkata Mohan, 2021). Signal peptides can guide target proteins into the correct secretory pathway during synthesis, thereby enhancing the efficiency of protein secretion (Kang et al., 2020). After screening signal peptides from 173 *Bacillus subtilis* strains, the signal peptide SP_YpuA_ was found optimal for *α*-amylase production, and the fermentation activity was increased by 1.28-fold (Li et al., 2022). Consequently, the optimization of signal peptides has emerged as a crucial strategy for enhancing enzyme production (Miao et al., 2021).

Different *Bacillus* strains can significantly affect the expression of foreign proteins. Some native proteases secreted by *Bacillus* can degrade the foreign proteins, thereby interfering or even completely terminating the expression of target proteins. In addition, *Bacillus* host strains also synthesize a large number of redundant proteins (Yu et al., 2015), which will compete with the secretion of target proteins to reduce the expression level. At present, a variety of protease-deficient *Bacillus* host strains have been constructed to increase the production of target enzymes, including *B. subtilis* 168, *Bacillus licheniformis* WX-02, and *B. amyloliquefaciens* HZ-12 (Osamura et al., 2023; Wu et al., 1991; Wong et al., 1994; Wei et al., 2015; Chen et al., 2022). This study aims to construct a high-expression engineered strain of amylase through strain screening, gene mining, and optimization of signal peptides and host strains. Furthermore, the enzymatic properties of the amylase were characterized, and its application potential in tobacco leaves was evaluated. This study provides a novel enzymatic resource for the development of amylases, demonstrating significant market potential and application value.

## 2 Materials and methods

### 2.1 Enzymes and chemical reagents

In this study, the 1.1 × S4 Fidelity polymerase chain reaction (PCR) Mix was purchased from Beijing Genesand Biotech Co., Ltd. (Beijing, China). T4 DNA ligase, XbaI, BamHI, and SamI were obtained from TransGen Biotech Co., Ltd. (Beijing, China). The ClonExpress II One Step Cloning Kit were get from Nanjing Vazyme Biotech Co., Ltd. (Nanjing, China). The Gel Extraction Kit and Plasmid Mini Kit I were purchased from Omega Bio-tek Co., Ltd. (Norcross, USA). Other chemical reagents were sourced from China National Pharmaceutical Group Chemical Reagent Co., Ltd. (Shanghai, China).

### 2.2 Construction of the recombinant expression strains

The methodology for constructing the recombinant expression strain followed established protocols from previous research(Chen et al., 2022). In this study, the construction of recombinant expression strain BAX-5/PT-17*amyE(WHC-115)* was presented as an example. According to the gene sequence of *amyE(WHC-115)* in *Bacillus subtilis* WHC-115, primers amyE-F and amyE-R were designed, and restriction sites of *Xba*I and *BamH*I were added at both ends of the primers, respectively. The genomic DNA of *B. subtilis* WHC-115 was used as a template to amplify the *amyE(WHC-115)* gene. Then, the recovered PCR product and the PT-17 vector were digested by restriction enzymes *BamH*I and *Xba*I. After purification, the digested PCR product and plasmid were linked by T4 DNA ligase at 4°C for 6–12 h. The ligation product was transformed into *E. coli* DH5*α* competent cells, and the single colony was verified by PCR amplification and gene sequencing. Finally, the free expression plasmid PT-17*amyE(WHC-115)* was successfully constructed.

The free expression plasmid PT-17*amyE(WHC-115)* was mixed with the BAX-5 competent cells. The cells were cultured for 3 h after electro-transformation, and spread on LB solid medium containing 20 μg/mL tetracycline. Finally, the positive single colony was verified by PCR amplification and gene sequencing. The other recombinant expression strains were performed using the same method. All the strains and plasmids constructed in this study were shown in Table 1. All the primers in this study were shown in Supplementary Table S1.

**Table 1:** Strains and plasmids used in this study.

Strains and plasmids	Relevant properties	Source
*Escherichia coli* DH5*α*	super44 Δ*lacU169* (f 80 lacZΔ*M15*) hsdR17recA1gyrA96thi1relA1	Stored in lab
*Bacillus amyloliquefaciens* LCCC10163	Wide-type	Stored in lab
*Bacillus subtilis* 168	Wide-type	Stored in lab
*Bacillus subtilis* SCK6	Wide-type	Stored in lab
*Bacillus licheniformis* WX-02	Wide-type	Stored in lab
*Bacillus amyloliquefaciens* HZ-12	Wide-type	Stored in lab
BAX-5	HZ-12 Δ*epr*Δ*nprE*Δ*aprE-a*Δ*aprX*Δ*mpr*	Stored in lab
BL10	WX-02 Δ*hag*Δ*mpr*Δ*vpr*Δ*ap*rXΔ*epr*Δ*bpr*Δ*wprA*Δ*aprE*Δ*amyL*Δ*bprA*	Stored in lab
*B.cereus* WHC-17	Wide-type	This study
*Bacillus subtilis* WHC-84	Wide-type	This study
*Bacillus subtilis* WHC-115	Wide-type	This study
*Bacillus velezensis* WHC-117	Wide-type	This study
BAX-5/PT-17*amyS(WHC-17)*	BAX-5 strain contains PT-17*amyS(WHC-17)* plasmid	This study
BAX-5/PT-17*amyE(WHC-84)*	BAX-5 strain contains PT-17*amyE(WHC-84)* plasmid	This study
BAX-5/PT-17*amyE(WHC-115)*	BAX-5 strain contains PT-17*amyE(WHC-115)* plasmid	This study
BAX-5/PT-17*amyE(WHC-117)*	BAX-5 strain contains PT-17*amyE(WHC-117)* plasmid	This study
BAX-5/pHY300P*amyA(LC)*	BAX-5 strain contains pHY300P*amyA(LC)* plasmid	This study
BAX-5/PT-17SP_001_*amyA(LC)*	BAX-5 strain contains PT-17SP_001_*amyA(LC)* plasmid	This study
BAX-5/PT-17SP_002_*amyA(LC)*	BAX-5 strain contains PT-17SP_002_*amyA(LC)* plasmid	This study
BAX-5/PT-17SP_003_*amyA(LC)*	BAX-5 strain contains PT-17SP_003_*amyA(LC)* plasmid	This study
SCK6/PT-17SP_003_*amyA(LC)*	SCK6 strain contains PT-17SP_003_*amyA(LC)* plasmid	This study
BL10/PT-17SP_003_*amyA(LC)*	BL10 strain contains PT-17SP_003_*amyA(LC)* plasmid	This study
168/PT-17SP_003_*amyA(LC)*	168 strain contains PT-17SP_003_*amyA(LC)* plasmid	This study
pHY300PLK	*Escherichia coli-Bacillus* shuttle vector; Ampr, Tetr	Stored in lab
PT-17	pHY300PLK contains Promoter P_43_ and terminator TamyL, Ampr, Tetr	Stored in lab
PT-17*amyS(WHC-17)*	PT-17 contains gene *amyS(WHC-17)*, Ampr, Tetr	This study
PT-17*amyE(WHC-84)*	PT-17 contains gene *amyE(WHC-84)*, Ampr, Tetr	This study
PT-17*amyE(WHC-115)*	PT-17 contains gene *amyE(WHC-115)*, Ampr, Tetr	This study
PT-17*amyE(WHC-117)*	PT-17 contains gene *amyE(WHC-117)*, Ampr, Tetr	This study
pHY300P*amyA(LC)*	pHY300PLK contains gene *amyA(LC)*, Ampr, Tetr	This study
PT-17SP_001_*amyA(LC)*	PT-17 contains Signal peptide SP_001_ and gene *amyA(LC)*, Ampr, Tetr	This study
PT-17SP_002_*amyA(LC)*	PT-17 contains Signal peptide SP_02_ and gene *amyA(LC)*, Ampr, Tetr	This study
PT-17SP_003_*amyA(LC)*	PT-17 contains Signal peptide SP_003_ and gene *amyA(LC)*, Ampr, Tetr	This study

### 2.3 Fermentation conditions of *α*-amylase

The strains were inoculated in LB liquid medium (10 g/L NaCl, 10 g/L tryptone, 5 g/L yeast extract, pH 7.2–7.4) as seed culture. The recombinant expression strains was added with 20 μg/mL of tetracycline in LB liquid medium. The seed cultures were incubated at 37°C and 180 r/min for 8–12 h. When the OD_600_ reached 3.5–4.0, the seed cultures (3%) were transferred into 50 mL fermentation medium (80 g/L tryptone, 25 g/L yeast extract, 5 g/L K_2_HPO_4_, and 6 g/L NH_4_Cl) at 37°C and 180 r/min for 48 h. In addition, the optimized components of the culture medium consisted of 65 g/L corn meal, 70 g/L soybean cake meal, 10 g/L NaCl, and 3 g/L CaCl_2_ for 52 h at 40°C and pH = 6, The volume of solution medium is 50 mL.

### 2.4 Detection of *α*-amylase activity

#### 2.4.1 Standard curve preparation

Glucose standard solutions were prepared at final concentrations of 0, 0.2, 0.4, 0.6, 0.8, 1.0, 1.2, 1.4, 1.6, 1.8, and 2.0 mg/mL (total volume: 40 μL per concentration). To each solution, 60 μL of 3,5-dinitrosalicylic acid (DNS)(Ghose method) reagent was added, followed by incubation at 99.9°C for 2 min. The samples were then rapidly cooled to 4°C, diluted with 100 μL deionized water in a 96-well plate, and the absorbance at 540 nm was measured. A standard curve was generated by plotting glucose concentration against the corresponding optical density (OD) values.

#### 2.4.2 Enzymatic activity assay

The reaction mixture contained: 10 μL phosphate buffer (45.23 g/L Na₂HPO₄·12H₂O, 8.07 g/L C₆H₈O₇·H₂O), 10 μL 4% (w/v) soluble starch solution and 20 μL crude enzyme extract. The mixture was incubated at 60°C for 5 min in a PCR thermocycler. Subsequently, 60 μL DNS reagent was added, and the samples were heated at 99.9°C for 2 min. After rapid cooling to 4°C, 100 μL deionized water was added, and absorbance was measured at 540 nm. Control: A blank was prepared by replacing the crude enzyme solution with 20 μL heat-inactivated enzyme, with all other steps identical.

#### 2.4.3 Calculations

The amount of reducing sugar released was quantified using a glucose standard curve. Enzyme activity is defined as: under given reaction conditions, 1 mL of enzyme solution reacts to produce the equivalent of 1 μmol of glucose per minute, which is regarded as one unit of enzyme activity, expressed in IU/mL. The formula is as follows: suppose the standard curve is y = kx + b. Then the sample enzyme activity:


$$(\mathrm{IU}/\mathrm{mL})=\frac{\frac{\left((\mathrm{Ae}-A0)-b\right)}{k*\mathrm{Df}*1000}}{\left(\mathrm{Wf}*T*V\right)}$$

Ae - absorbance value of the enzyme solution reaction of the sample.
A0 -absorbance value of blank control.
Df - dilution of the enzyme solution of the sample.
Relative molecular weight of WF-glucose (180.16).
T-enzyme reaction time (5 min).
V - Enzyme volume (20 μL).

### 2.5 Enzyme characterization

After fermentation for 48 h, the fermentation broth was collected and centrifuged at a rotational speed of 12,000 r/min for 10 min. Then, 5 mL supernatant was taken into the 30 kDa ultrafiltration tube, which was centrifuged at 2000 r/min for 30 min to obtain the *α*-amylase liquid with higher concentration. The enzyme solution after ultrafiltration will be used for enzymatic properties analysis.

The diluted enzyme solution was subjected to reaction at 40, 50, 60, 70, 80, 90°C, respectively. After that, the enzyme activity of different treatment groups was detected. The enzyme activity was determined by DNS method to determine the optimal reaction temperature, except for the reaction temperature, the reaction system and reaction conditions are the same as the method for determining enzyme activity in 2.4 section.

The enzyme solution after dilution was placed in water bath at 30, 40, 50, 60, 70, 80, 90°C for 30 min. After that, the enzyme activity of different treatment groups were detected. The amylase activity was determined by DNS method, the method is the same as that for determining enzyme activity in 2.4 section in this article.

The enzyme solution was diluted in FeCl_2_, CaCl_2_, NaCl, MgCl_2_, KCl and CuCl_2_ with a metal ion concentration of 5 mmol/L, and then the diluents were left for 30 min. After that, the enzyme activity of different treatment groups were detected. Then the amylase activity was determined by DNS method (the enzyme diluents without metal ions were used as the control group). The determination method is the same as that for determining enzyme activity in section 2.4 in this article.

The enzyme solution was diluted in phosphate buffers at pH 4.0, 5.0, 6.0, 7.0, 8.0, 9.0, respectively, and placed at room temperature for 30 min. The enzyme activity of the enzyme solutions at different pH buffers were detected by DNS method, the reaction system and reaction conditions are the same as the method for determining enzyme activity in 2.4 part.

The solutions mentioned above used for preparing solutions with different pH gradients are phosphate buffer solutions, they are the same as the phosphate buffer liquid used in the detection of amylase activity by DNS method in section 2.4, and the basic preparation method of this buffer solution has been mentioned in section 2.4 of this article.

### 2.6 Enzyme treatment and evaluation of tobacco samples

The fermentation broth was concentrated by ultrafiltration and sprayed on B4F tobacco leaves from Chongqing. The concentrated enzyme solution was sprayed with 7 U enzyme activity per gram of tobacco leaf. Then, the tobacco leaves were treated at 45°C and 75% humidity for 48 h, and the tobacco leaves treated with enzymes were inactivated at 100°C in the drying box for 30 s. The treated tobacco leaves were cut into shredded tobacco with a chopper, and the cut tobacco was evenly packed into the 84 mm empty smoke pipe. Then, seven experts authorized by the China National Tobacco Corporation evaluated the sensory quality of cigarettes.

The pyrolysis of tobacco ingredients was used by pyrolysis instrument, gas chromatograph and mass spectrometer (GC–MS). The tobacco leaves treated with enzymes were turned into powder through the blender mechanism, and 1 mg of powder was weighed into the pyrolysis tube. The pyrolysis tube was then sent into a pyrolyzer, each component entered the GC–MS system through the transmission line. The sample was heated to 900°C at a rate of 50°C/s and maintained at this temperature for 5 s. The column model used was DB-WAX capillary column (60 m × 0.25 mm × 0.25 μm), and the carrier gas was helium with a flow rate of 1.5 mL/min. The injection port temperature was 250°C. The column temperature program was as follows: initial temperature of 40°C held for 1 min, and then ramped at 5°C/min to 200°C and the temperature then rose to 250°C at a rate of 10°C/min and remained at this temperature for 20 min.

### 2.7 Statistical analysis

SPSS 26.0 was used for statistical analysis, including calculation of the mean and deviation values. Origin 2017 and Prism 8.0 was used for data processing and making histograms.

## 3 Results

### 3.1 Expression of different *α*-amylase genes and analysis of the *α*-amylase Amy (LC)

Research has found that *Bacillus* spp. is an important source of amylase genes (Ye et al., 2024). Therefore, based on the existing *Bacillus* spp. in the laboratory, we successfully amplified five different alpha-amylase gene fragments from wild strains *B. cereus* WHC-17, *B. subtilis* WHC-84, *B. subtilis* WHC-115, *B. velezensis* WHC-117, and *B. amyloliquefaciens* MK10163, respectively. Finally, 5 different *α*-amylase genes from WHC-17, WHC-84, WHC-115, WHC-117 and MK10163 were successfully amplified (Gel electrophoresis images of gene fragments are shown in the Supplementary Figure S1). The corresponding *α*-amylase gene fragments were labeled as *amyS(WHC-17)*, *amyE(WHC-84)*, *amyE(WHC-115)*, *amyE(WHC-117)*, and *amyA(LC)*. The gene fragments were further sequenced by Beijing Tsingke Biotechnology Co., Ltd. to obtain the gene sequence information (shown in Supplementary material 1).

**Figure 1:**
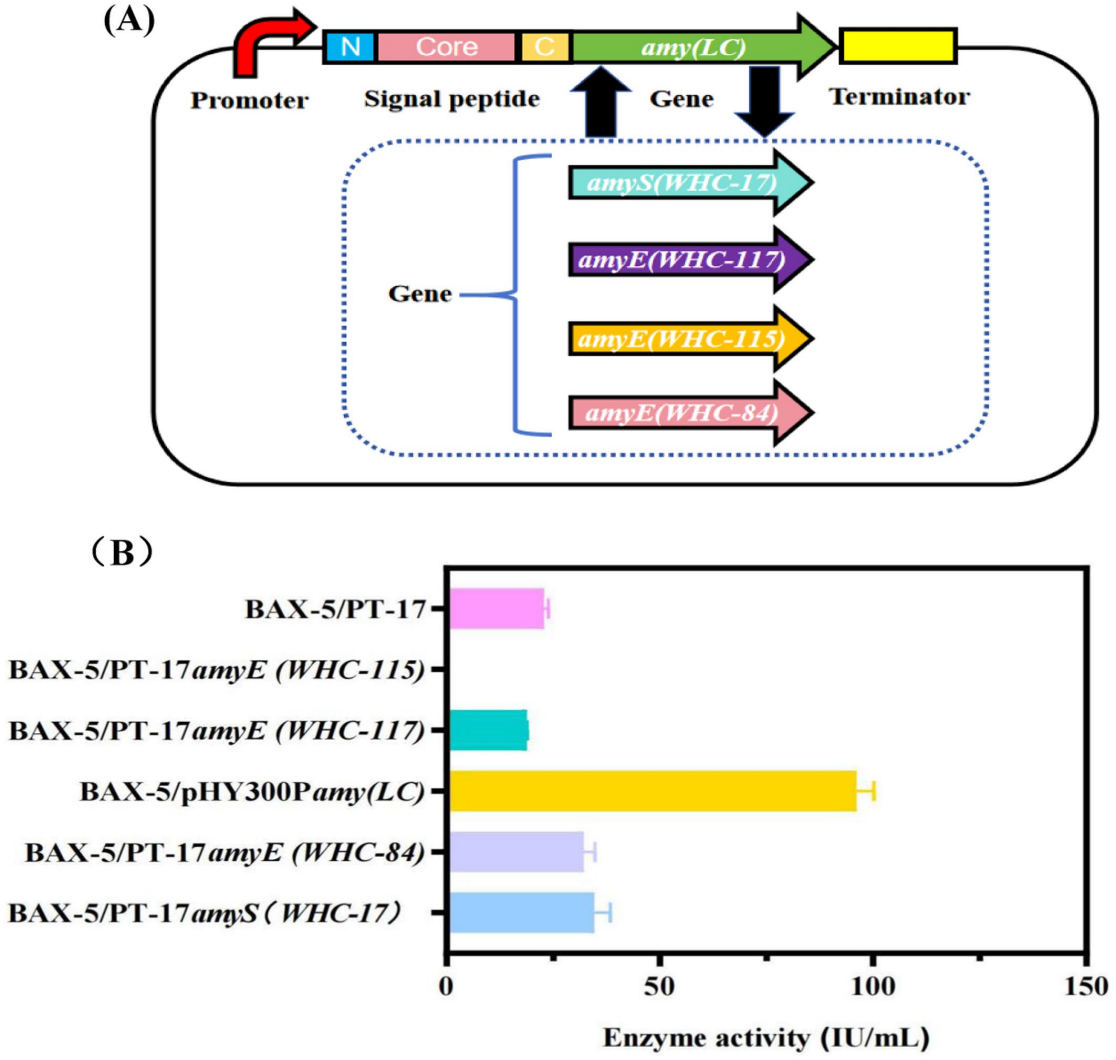
Enzyme activities of different *α*-amylase gene expression strains and analysis of the *α*-amylase Amy (LC). **(A)** Construction of different gene expression vectors. **(B)** Comparison of extracellular amylase activity of recombinant strains with different amylase genes.

Subsequently, the *α*-amylase gene fragments of *amyS(WHC-17)*, *amyE(WHC-84)*, *amyE(WHC-115)*, *amyE(WHC-117)* and *amyA(LC)* were used to construct 5 expression plasmids, which were transformed into BAX-5 to obtain the recombinant expression strains, including BAX-5/PT-17*amyS(WHC-17)*, BAX-5/PT-17*amyE(WHC-84)*, BAX-5/PT-17*amyE(WHC-115)*, BAX-5/PT-17*amyE(WHC-117)*, and BAX-5/pHY300P*amyA(LC)*. Through further fermentation analysis, the *α*-amylase activities of the engineered strains were shown in Figure 1B. The *α*-amylase activity (96.09 IU/mL) of BAX-5/pHY300P*amyA(LC)* was much higher than that of other engineered strains. The enzyme activity of strain BAX-5/PT-17*amyS(WHC-17)* and BAX-5/PT-17*amyE(WHC-84)* were only 34.64 IU/mL and 32.15 IU/mL, respectively, which were slightly higher than that of engineered strain BAX-5/PT-17 (22.85 IU/mL). The enzyme activity of BAX-5/PT-17*amyE(WHC-117)* was lower than that of BAX-5/PT-17, and strain BAX-5/PT-17*amyE(WHC-115)* even lost *α*-amylase activity. Therefore, the *α*-amylase gene *amyA(LC)* was selected for further analysis and expression optimization.

### 3.2 Effects of different signal peptides on *α*-amylase expression

Signal peptides are one of the critical elements influencing the level of gene expression, and the signal peptide part of *amy (LC)* gene was predicted and analyzed by using the signal peptide prediction tool (https://novopro.cn/tools/signalp). The original signal peptide of Amy (LC) amylase belongs to the SP (Sec/SPI) type signal peptide, and the signal peptidase binding site is between 31 and 32 (shown in Supplementary Figure S3). Based on the signal peptide database SPSED (http://www.spsed.com), this study screened the optimal signal peptides for the amylase gene, included three signal peptides SP_001_, SP_002_ and SP_003_. In this study, those signal peptides SP_001_, SP_002_, and SP_003_, were fused with the gene *amyA(LC)* and the PT17 vector to obtain three recombinant vectors PT-17SP_001_*amyA(LC)*, PT-17SP_002_*amyA(LC)* and PT-17SP_003_*amyA(LC)*, respectively. All the recombinant plasmids were further transformed into the host strain BAX-5 to obtain the engineering strains BAX-5/PT-17SP_001_*amyA(LC)*, BAX-5/PT-17SP_002_*amyA(LC)*, and BAX-5/PT-17SP_003_*amyA(LC)*, respectively.

**Figure 2:**
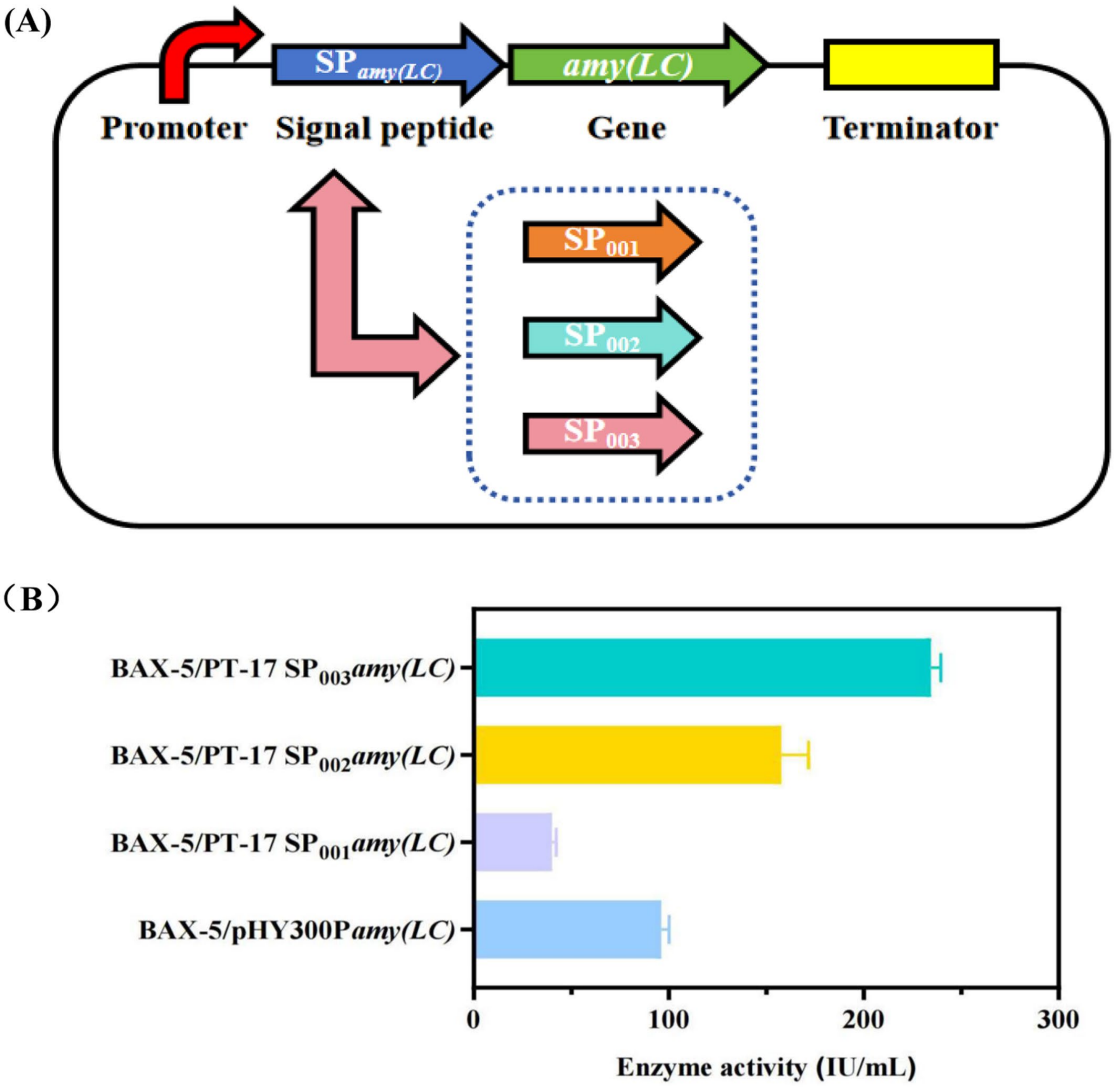
Effects of different signal peptides on the enzyme activities of *α*-amylase Amy (LC) **(A)** Construction of different signal peptides expression vectors. **(B)** Comparison of extracellular amylase activity of recombinant strains with different signal peptides.

The constructed engineered strains containing different signal peptides were fermented for *α*-amylase analysis, and the fermentation activities were shown in Figure 2B, these three signal peptides led to significantly different *α*-amylase activities. Therein, the *α*-amylase activities by SP_003_ reached 234.52 IU/mL, which was much higher than that of SP_001_ (40.06 IU/mL) and SP_002_ (143.18 IU/mL). In summary, the fermentation activity of *α*-amylase was further improved by optimizing the signal peptide, and the signal peptide SP_003_ was optimal for mediating the expression of gene *amyA(LC)*.

### 3.3 Effects of different host strains on *α*-amylase expression

**Figure 3:**
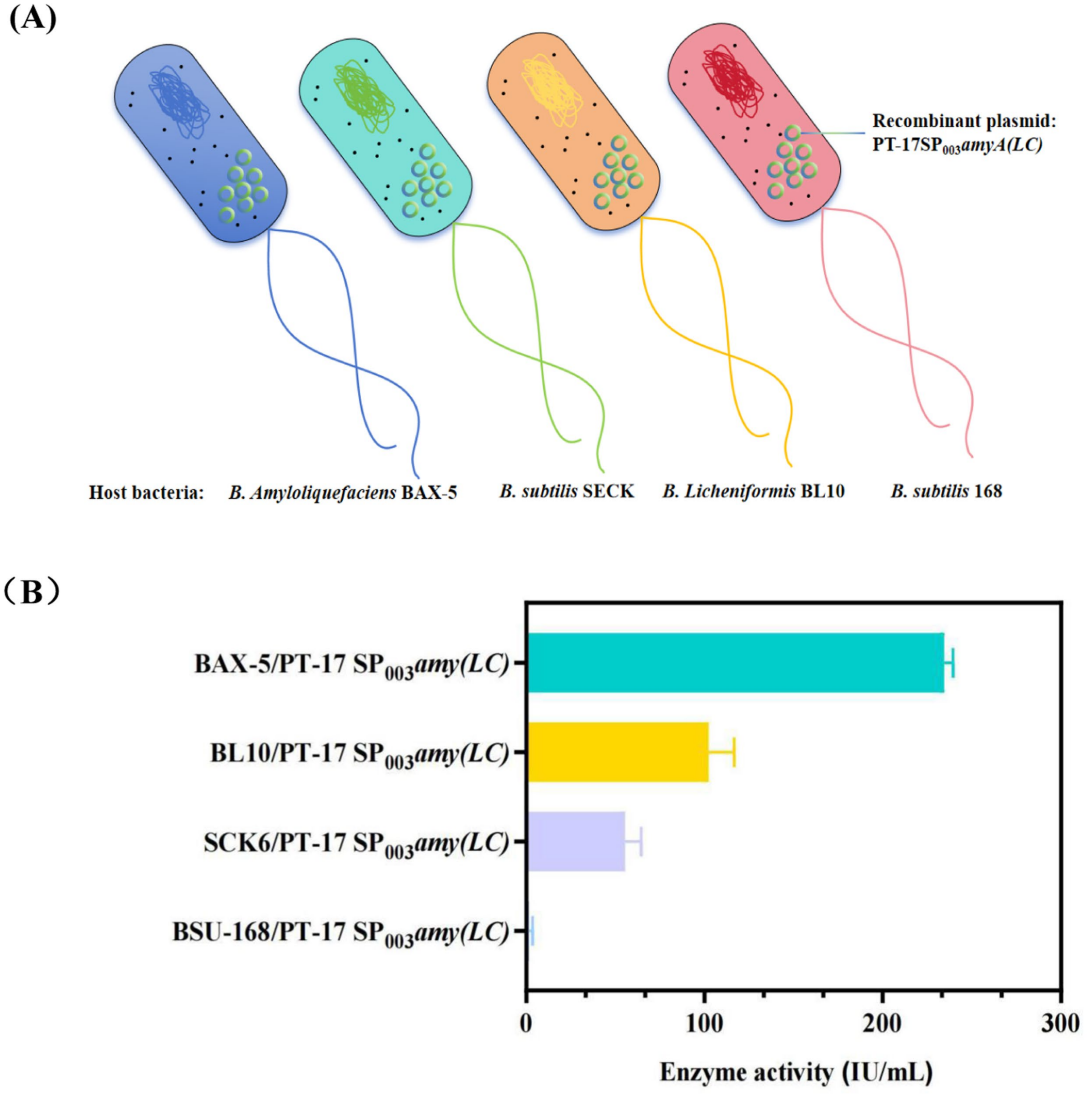
Effects of different host bacteria on the enzyme activities of *α*-amylase Amy (LC) **(A)** Construction of recombinant strains with different host bacteria. **(B)** Comparison of extracellular amylase activity of recombinant strains with different host bacteria.

In addition, this study further screened the host strains, including *B. subtilis* SCK6, *B. subtilis* 168, and *B. licheniformis* BL10 (deficient in 10 genes on the basis of *Bacillus licheniformis* WX-02)(Wei et al., 2015). Accordingly, three new engineered strains were obtained, named as SCK6/PT-17SP_003_*amyA(LC),* 168/PT-17SP_003_*amyA(LC)* and BL10/PT-17SP_003_*amyA(LC)*. As it showed in Figure 3B. The result showed that the enzyme activity of engineered strain BAX-5/PT-17SP_003_*amyA(LC)* was the highest, which was significantly higher than that of the other engineered strains. Therefore, the *B. amyloliquefaciens* BAX-5 was the optimal host bacteria.

### 3.4 Characterization of enzymatic characterization of recombinant expression strain

**Figure 4:**
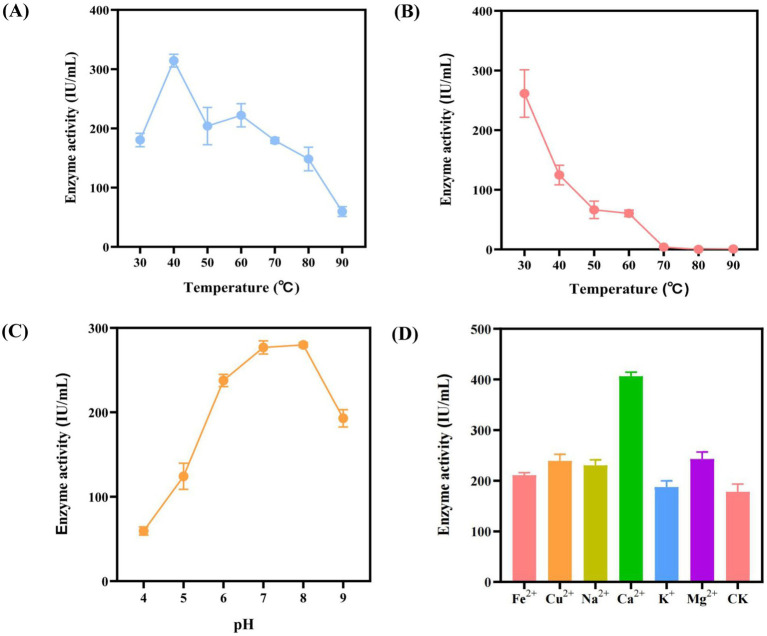
Enzyme characterization. **(A)** Reaction temperature: The ability of the enzyme to degrade substrates at different reaction temperatures were evaluated. **(B)** Thermal stability: The tolerance of the enzyme to different temperature environments was evaluated. **(C)** pH stability: The tolerance of the enzyme to different pH environments was evaluated. **(D)** Effects of metal ions on *α*-amylase activity. CK: negative control, no metal ions added.

In the previous stage, this study obtained the optimal *Bacillus* expression system for the target enzyme genes. In order to further understand the characteristics of the amylase, the amylase activity at different reaction temperatures, temperature tolerance, pH tolerance, metal ion tolerance and organic solvent tolerance were analyzed. Firstly, the amylase reaction temperature was adjusted to 30°C, 40°C, 50°C, 60°C, 70°C 80°C and 90°C, respectively, and then the amylase activities of different temperature groups were measured by DNS method. The results are shown in Figure 4A, the amylase activity exhibited its highest activity at 40°C, reaching 314.37 IU/mL. Then, the amylase activity values gradually decreased as the temperature gradually increased above 40°C. When the reaction temperature reached 90°C, the amylase activity was only 59.65 IU/mL. Subsequently, the results of the temperature tolerance analysis indicated a decreasing trend with the increase of temperature at the range of 30°C to 90°C (Figure 4B). Among them, the highest amylase activity reached 261.39 IU/mL after being placed at 30°C for 30 min. When the enzyme solution was placed at 70°C, 80°C or 90°C, almost no amylase activity was detected. Therefore, *α*-amylase Amy (LC) does not possess high temperature tolerance.

Furthermore, this study investigated the effect of pH on amylase activity. The enzyme solution was exposed to different pH for 30 min, and the results of enzyme activity are shown in Figure 4C. The enzyme activity reached the peak of 279.92 IU/mL under pH 8. As the pH increased or decreased to both sides, the enzyme activity gradually decreased. As shown in Figure 4D, the performance of enzyme activity varies greatly under different metal ion environments. Compared with the control group, the k^+^ had almost no effect on the amylase activity, while the Fe^2+^, Na^2+^, Cu^2+^, Mg^2+^ metal ions leading to a slight increase in the amylase activity. In addition, the strongest effect on the enzyme activity was observed in the Ca^2+^, which nearly doubled the amylase activity compared with the control group.

### 3.5 Replacement of culture medium for recombinant strain

**Figure 5:**
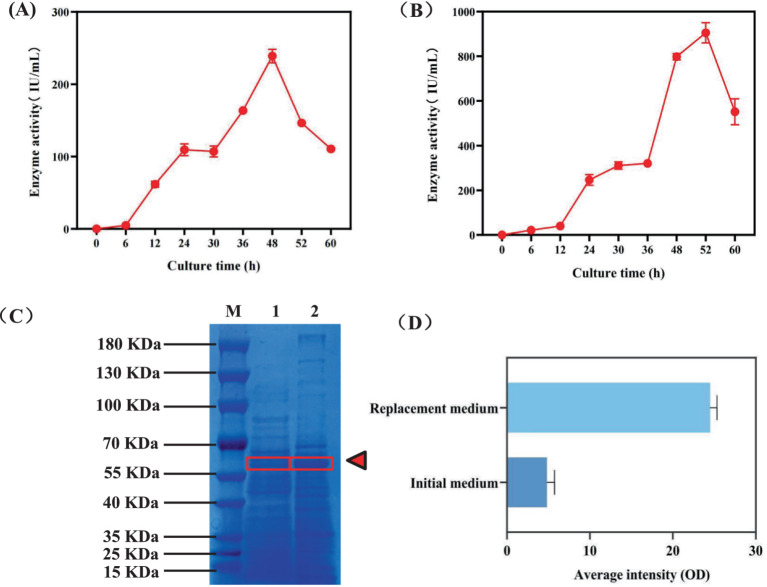
Comparison of the expression ability of *α*-amylase Amy (LC) before and after medium replacement. **(A)** Changes in enzyme activity over time when *α*-amylase Amy (LC) was fermented in the original medium. **(B)** The change of enzyme activity with time when *α*-amylase Amy (LC) was fermented in replaced medium. **(C)** Changes in *α*-amylase Amy (LC) expression before and after medium replacement. Sample 1: Expression of *α*-amylase Amy (LC) after fermentation of the previous medium. Sample 2: Expression of *α*-amylase Amy (LC) after fermentation with replaced medium.

To better understand the fermentation status of the engineered strain BAX-5/PT-17SP_003_*amyA(LC)* during the fermentation period, this study determined the amylase activity of the BAX-5/PT-17SP_003_*amyA(LC)* at different time over the fermentation period. As shown in Figure 5A, the amylase activity gradually grew form 0–48 h, and the enzyme activity reached its peak at 238.91 IU/mL at 48 h. Subsequently, the amylase activity began to decline after 48 h. Therefore, the optimal fermentation duration for the amylase in this study was determined to be 48 h.

Then, this study attempted to further enhance the *α*-amylase activity by replacing the culture medium. The strain BAX-5/PT-17SP_003_*amy(LC)* was used to fermentation with a known medium (The mixture consisted of 65 g/L corn meal, 70 g/L soybean cake meal, 10 g/L NaCl, and 3 g/L CaCl_2_ for 52 h at 40°C and pH 6, The volume of solution medium is 50 mL) that highly expressed *α*-amylase of engineered *Bacillus*. As shown in Figure 5B, the *α*-amylase activity increased with the extension of fermentation time. The optimal fermentation time was 52 h, and the *α*-amylase activity reached 904.91 IU/mL, which was nearly 4 times as much as that of the original culture medium. Then, the SDS-PAGE result showed that the *α*-amylase expression level was significantly improved after replacement (Figure 5C). The expression intensity of *α*-amylase Amy (LC) in SDS-PAGE gel map was analyzed. As shown in Figure 5D, the average intensity of *α*-amylase in the initial medium was 4.81, and the average intensity of *α*-amylase was 24.49 after replacement of culture medium, which was 5.09 times than that of the original medium. This result suggests that the increase in *α*-amylase activity may be due to the enhanced expression level of *α*-amylase.

### 3.6 Application of amylase in tobacco leaves

**Figure 6:**
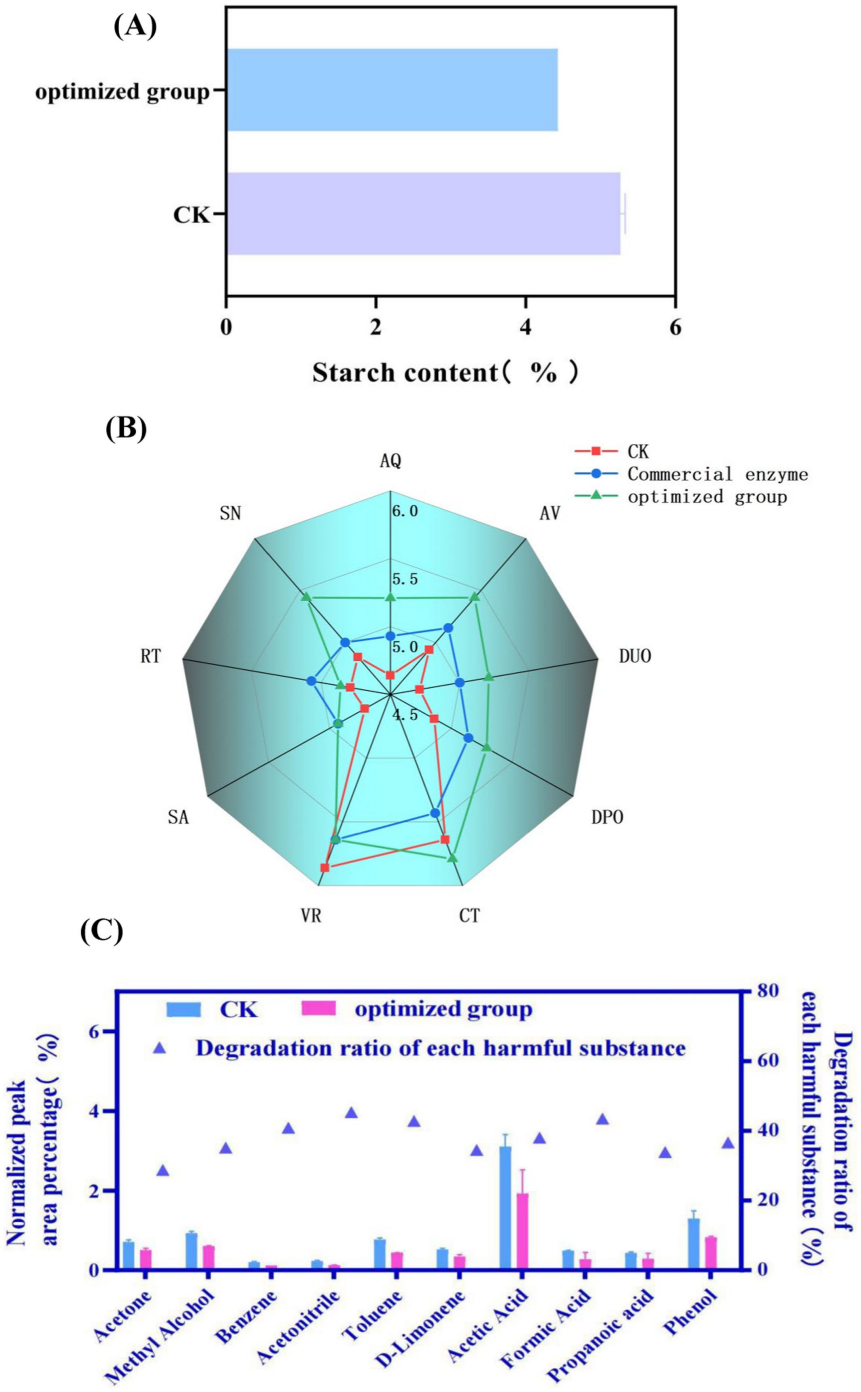
Quality assessment of tobacco treated with amylase amy (LC). **(A)** The effect of enzyme treatment on starch content. CK: control group. **(B)** Sensory evaluation of tobacco smoking during combustion. (Evaluation is conducted from 9 different dimensions. AQ, aroma quality; AV, aroma volume; DUO, decrease of unpleasant odor; DPO, decrease of pungent odor; CT, concentration; VR, vigorous; SA, sweete aroma; RT, remaining taste; SN, softness). **(C)** The content of harmful substances released during the pyrolysis process of tobacco leaves. The content of harmful substances is depicted by a bar chart (left y-axis), while the percentage reduction of these substances in the enzyme-treated group compared to the control group is represented by a blue triangle (right y-axis). CK: control group.

Based on the aforementioned research findings, the strain BAX-5/PT-17*SP_003_amy(LC)* was further applied to the treatment of tobacco leaves, and the quality of cigarettes made from treated tobacco leaves was evaluated. This study determined the performance of each group of the tobacco leaves treated with enzymes after preparation into cigarettes, and the degradation of harmful substances in tobacco by *α*-amylase was studied by Pyrolysis Gas Chromatography–Mass Spectrometry (Py-GC/MS). As shown in Figure 6A, the starch content in fermented tobacco leaves decreased from 5.27 to 4.43% compared with the control group. Subsequently, the application potential of the amylase was further evaluated.

Next, the tobacco leaves treated with enzymes are prepared into cigarettes. The prepared cigarettes were used for sensory evaluation. As shown in Figure 6B, most of the indexes of the cigarettes fermented by the enzyme solution produced by the engineered bacteria BAX-5/PT-17SP_003_ *amy(LC)* (the optimized group) were better than those of the control group and the commercial enzyme group. The improvement of aroma quality was more significant than that in the control group and commercial enzyme group. The index of the optimized group was 5.21, while the score of the control group and commercial enzyme group was 4.64 and 5.07, respectively. The performance of the optimized group was better than that of the commercial enzyme group and the control group in terms of aroma quantity, softness degree, decrease of unpleasant odor, decrease of pungent odor, concentration but the vigorous score of the optimized group was lower than that of the control group. Although the sweet aroma score of the optimized group was higher than that of the control group, the performance was the same as that of the commercial enzyme group. The remaining taste index of the optimized group was higher than that of the control group, but lower than that of the commercial enzyme group.

Finally, the tobacco powder prepared from treated tobacco leaves was tested for Py/GC–MS experiment. The method simulates the environment of tobacco combustion, and the ratio of the peak area of each substance to the total peak area was used to characterize the change of substance content. As shown in Figure 6C, the peak area proportion of ten common harmful substances in the fermented tobacco leaves decreased compared with the blank sample, and the reduction rates for all ten substances ranged from 28.74 to 45.93%. Among them, the reduction rate of benzene, acetonitrile, toluene and formic acid is more than 40%, the reduction rates of phenol, propionic acid, D-limonene, acetic acid and methanol were between 30 and 40%. The degradation rate of acetone was the lowest, only 28.74%.

In summary, the *α*-amylase Amy (LC) enzyme liquid obtained by the engineered strain BAX-5/PT-17SP_003_*amyA (LC)* can not only reduce the starch content in tobacco leaves, but also significantly improve the sensory quality of cigarettes and it can reduce the proportion of ten harmful substances in the total substances during cigarette combustion. Therefore, the engineered strain BAX-5/PT-17SP_003_*amyA (LC)* has great significance for the application of tobacco leaf enzyme treatment in future industrial production.

## 4 Discussion

The starch content of tobacco plays a crucial role in the harmful substances and quality of tobacco. In order to reduce the influence of starch content on the tobacco and improve the quality of tobacco leaves, this study aimed to develop a novel *α*-amylase for tobacco starch degradation through gene mining, signal peptide and host strain optimization.

Firstly, the *α*-amylase gene fragments of 5 strains were amplified and sequenced. The repetitive *α*-amylase genes were removed by sequence alignment, and 5 *α*-amylase genes with significantly different sequences were successfully excavated. Then, above 5 *α*-amylase genes were used as target genes to construct different free expression vectors, which were further transformed into the host strain BAX-5 to construct the recombinant expression strains. The *α*-amylase fermentation activities were analyzed. It is worth noting that BAX-5/PT-17*amyE(WHC-115)* and BAX-5/PT-17*amyE(WHC-117)* showed lower enzyme activity than the control group. This is because, although the gene sequences of different *α* -amylases are similar, even very small differences in base sequences can affect the three-dimensional structure of the enzymes, especially when key amino acids change, it often significantly affects the activity of enzymes. Li et al. also reported similar findings: when the Ala269 residue in sucrose-6-phosphate hydrolase (SacA) was mutated to Thr269, the enzymatic activity of the Ala269Thr mutant increased by approximately 50%. This demonstrates that even a single amino acid mutation can significantly alter enzyme activity (Li et al., 2025). Due to the best enzymatic activity performance, the gene *amyA(LC)* was finally determined as the optimal *α*-amylase gene.

The signal peptide is a 5–30 amino acid part at the front of a protein, which is important for extracellular secretion of target protein (Smets et al., 2022). Various signal peptides from different strains usually resulted in different expression levels for target protein (Liu et al., 2022), and replacement of signal peptides has been an important optimization strategy to enhance protein expression. Since *α*-amylase is extracellular protein, the signal peptide will play an important role in its secretion. To further improve the expression of *α*-amylase, the signal peptides of *α*-amylase were optimized, and the DNA sequences of signal peptides were mined from the signal peptide library. The original signal peptide coding region of gene *amyA(LC)* was replaced, and the signal peptide with the strongest effect on *α*-amylase was screened. Both the *α* -amylases mediated by SP_002_ and SP_003_ showed high enzymatic activities, among which SP_003_ exhibited the highest *α*-amylase activity. These three signal peptides (SP_001,_ SP_002_ and SP_003_) were derived from the gene *tag1* of *Lactobacillus plantarum* (Mathiesen et al., 2009), genes *bglS* and *dacB* of *B. subtilis* (Yao et al., 2019; Liu et al., 2019), respectively. It indicated that the signal peptides from *Bacillus* were more beneficial for expression of gene *amyA(LC)*. Ultimately, the signal peptide SP_003_, derived from the *dacB* gene of *B. subtilis* 168 (Liu et al., 2019) was confirmed to be the optimal signal peptide for mediating the expression of gene *amyA(LC)*.

Different *Bacillus* host strains can significantly affect the expression of foreign proteins (Osamura et al., 2023; Wu et al., 1991; Wong et al., 1994; Wei et al., 2015). Therefore, the plasmid PT-17SP_003_*amyA (LC)* was further transformed into several host strains, including *B. amyloliquefaciens* BAX-5, *B. subtilis* SCK6, *B. subtilis* 168, and *B. licheniformis* BL10. Among them, the BAX-5/PT-17SP_003_*amyA(LC)* showed the highest *α*-amylase activity. Therefore, *B. amyloliquefaciens* BAX-5 is considered to be the most suitable host strain for *amyA (LC)* expression. *B. amyloliquefaciens* BAX-5 was obtained by knocking out five protease genes *mpr*, *epr*, *nprE*, *aprE* and *aprX* of wild-type *Bacillus amyloliquefaciens* HZ-12. This five genes were classified as important intracellular or extracellular protease genes in strain HZ-12. The deletion of these five protease genes might reduce the secretory pressure by the corresponding proteins on the host bacteria *B. amyloliquefaciens* BAX-5 (Chen et al., 2022), thereby increasing the secretion of exogenous *α*-amylase.

The enzymatic properties of biological enzymes can better reflect their characteristics. In this study, through enzymatic property analysis experiments, we found that amylase Amy (LC) was a medium temperature neutral amylase, and its tolerance to weak acidic environment was stronger than that to weak alkali environment, and calcium ion environment helped to enhance its enzymatic activity. This may be attributed to the presence of numerous binding sites within the structure of *α*-amylase that can interact with calcium ions. By binding with calcium ions, the structural stability of *α*-helix in the three-dimensional structure of *α*-amylase can be increased, so as to further improve the stability of the entire enzyme structure and stabilize the *α*-amylase activity (Abedi et al., 2024).

We further enhanced the activity of *α*-amylase Amy (LC) by using a laboratory-available medium formulation. The replaced medium contains soybean cake flour, a nitrogen source commonly used in industrial fermentation, and corn flour, a carbon source commonly used, as well as two inorganic salts: NaCl and CaCl_2_. Compared with the new medium, the main components of initial medium were yeast extract, tryptone, NH_4_Cl and K_2_HPO_4_, in which yeast extract, and tryptone acted as organic nitrogen sources, and NH_4_Cl was inorganic nitrogen sources. Although the first two substances (tryptone and yeast extract) have a small amount of carbon source components, the medium still lacks components that can be used as the main carbon source. Therefore, the main nutrients provided by the replaced medium are more comprehensive than the initial medium, which may be the main reason why the new medium is better than the initial medium. In addition, starch and other carbon sources contained in corn meal can induce the synthesis of amylase (Mishra and Behera, 2008; Ensari et al., 1995). The initial medium could not continuously improve the enzyme production efficiency through this induction mechanism. Moreover, the calcium ions in the inorganic salt can stabilize the enzyme activity in the optimized medium (Zhao et al., 2024). Finally, the cost of each component in the replaced medium is lower than the initial medium, which means that the new medium is more suitable for large-scale applications in industrial production in the future.

After medium replacement, we used the enzyme liquid after ultrafiltration to deal with tobacco leaves and evaluated the results of treated tobacco leaves. The results of sensory evaluation showed that most of the sensory indexes of cigarettes prepared by amylase Amy(LC) treated tobacco were better than those prepared by commercial enzyme fermented tobacco and control group. Reducing sugar is an important product of starch hydrolysis. Along with the degradation of starch, the content of reducing sugar increases accordingly, and sugar has a significant impact on the quality of tobacco leaves. On the one hand, sugar can be transformed into aromatic substances through reactions such as the Maillard reaction and caramelization reaction. On the other hand, an increase in sugar content can improve sweetness. This might explain the improvement in indicators such as the aroma quality, aroma quantity and sweetness of cigarettes prepared from enzyme-treated tobacco leaves in the sensory evaluation results (Yang et al., 2014). In addition, the amylase preparation from *B. amyloliquefaciens* strain isolated from tobacco was used to prepare flue-cured tobacco, and the large molecular substances such as starch in the flue-cured tobacco also showed a significant reduction. At the same time, the aroma quality and aroma quantity of the flue-cured tobacco were enhanced (Gong et al., 2023). This is consistent with the results of this study.

Since the incomplete combustion of starch in tobacco will also affect the release of harmful substances. Therefore, we also analyzed the release of harmful substances when tobacco was burned after enzyme treatment through thermal cracking experiments. The result indicated that the proportion of these ten harmful substances had decreased. Although there are limited reports on how these harmful substances are formed, But the 10 substances reduced after enzyme treatment were on the list of 149 harmful substances produced during the burning of tobacco (Rodgman and Green, 2003). All of these substances are toxic, carcinogenic and addictive to some degree. It can be seen that cigarettes made from tobacco leaves treated by Amy (LC) enzyme have certain positive effects on improving sensory quality and reducing harmful substances.

The above results and conclusions preliminarily prove that the engineered strain BAX-5/PT-17SP_003_*amyA(LC)* has great significance for reducing the damage and increasing the quality of tobacco leaves. It has great application potential in the enzyme treatment step of tobacco production.

## Data Availability

The data presented in this study have been deposited in the NCBI repository, with accession numbers: PV799417 (The gene sequence of amyS (WHC-17)), PV799418 (The gene sequence of amyE (WHC-84)), PV799419 (The gene sequence of amyE (WHC-115)), PV799420 (The gene sequence of amyE (WHC-117)), PV799421 (The gene sequence of amyA (LC)).

## References

[ref1] Abedi E. Kaveh S. Hashemi S. M. B. (2024). Structure-based modification of a-amylase by conventional and emerging technologies: comparative study on the secondary structure, activity, thermal stability and amylolysis efficiency. Food Chem. 437:137903. doi: 10.1016/j.foodchem.2023.137903, PMID: 37931423

[ref2] Cai M.-Z. Kee P. E. Ng H. S. Chen P.-T. (2022). Development of *Bacillus subtilis* self-inducible expression system for keratinase production using piggery wastewater. J. Taiwan Inst. Chem. Eng. 137:104218. doi: 10.1016/j.jtice.2022.104218

[ref3] Chen W. Li L. Ye C. Zhao Z. Huang K. Zou D.. (2022). Efficient production of extracellular alkaline protease in *Bacillus amyloliquefaciens* by host strain construction. Lwt 163:113620. doi: 10.1016/j.lwt.2022.113620

[ref4] Chen X. Liu L. Zhang Y. Zhou X. Lin T. Song Y.. (2017). Acetylsalicylic acid application decreased tobacco-specific nitrosamines and its precursors but maintained quality of air-cured burley tobacco (*Nicotiana tabacum* L.). Ind. Crop. Prod. 104, 221–228. doi: 10.1016/j.indcrop.2017.04.031

[ref5] Chu K. L. Koley S. Jenkins L. M. Bailey S. R. Kambhampati S. Foley K.. (2022). Metabolic flux analysis of the non-transitory starch tradeoff for lipid production in mature tobacco leaves. Metab. Eng. 69, 231–248. doi: 10.1016/j.ymben.2021.12.003, PMID: 34920088 PMC8761171

[ref6] Ensari A. N. Y. Otludil B. Aytekin A. M. C. (1995). Effect of starch induced bacterial growth and amylase production in *Bacillus subtilis*. Starch 47, 315–321. doi: 10.1002/star.19950470807

[ref7] Gong Y. Li J. Deng X. Chen Y. Chen S. Huang H.. (2023). Application of starch degrading bacteria from tobacco leaves in improving the flavor of flue-cured tobacco. Front. Microbiol. 14:1211936. doi: 10.3389/fmicb.2023.1211936, PMID: 37440887 PMC10335769

[ref8] Kang X.-M. Cai X. Huang Z.-H. Liu Z.-Q. Zheng Y.-G. (2020). Construction of a highly active secretory expression system in *Bacillus subtilis* of a recombinant amidase by promoter and signal peptide engineering. Int. J. Biol. Macromol. 143, 833–841. doi: 10.1016/j.ijbiomac.2019.09.144, PMID: 31765756

[ref9] Li X. Shi S. Hao Y. Zhai Z. Zhao Z. Feng X.. (2025). Surface hydrophilic amino acids of sucrose-6-phosphate hydrolase SacA play a key role in high acid production rates in *Lacticaseibacillus casei*. Lwt 218:117465. doi: 10.1016/j.lwt.2025.117465

[ref10] Li H. Yao D. Ying J. Han X. Zhang X. Fang X.. (2022). Enhanced extracellular raw starch-degrading *α*-amylase production in *Bacillus subtilis* through signal peptide and translation efficiency optimization. Biochem. Eng. J. 189:108718. doi: 10.1016/j.bej.2022.108718

[ref11] Liu P. Guo J. Miao L. Liu H. (2022). Enhancing the secretion of a feruloyl esterase in *Bacillus subtilis* by signal peptide screening and rational design. Protein Expr. Purif. 200:106165. doi: 10.1016/j.pep.2022.106165, PMID: 36038098

[ref12] Liu Q. Li R. Shi H. Yang R. Shen Q. Cui Q.. (2023). A recombineering system for *Bacillus subtilis* based on the native phage recombinase pair YqaJ/YqaK. Eng. Microbiol. 3:100099. doi: 10.1016/j.engmic.2023.100099, PMID: 39628932 PMC11610992

[ref13] Liu Y. Shi C. Li D. Chen X. Li J. Zhang Y.. (2019). Engineering a highly efficient expression system to produce Bcapro protease in *Bacillus subtilis* by an optimized promoter and signal peptide. Int. J. Biol. Macromol. 138, 903–911. doi: 10.1016/j.ijbiomac.2019.07.175, PMID: 31356949

[ref14] Mathiesen G. Sveen A. Brurberg M. B. Fredriksen L. Axelsson L. Eijsink V. G. (2009). Genome-wide analysis of signal peptide functionality in *Lactobacillus plantarum* Wcfs1. BMC Genomics 10, 1–13. doi: 10.1186/1471-2164-10-425 19744343 PMC2748100

[ref15] Miao H. Jiang R. Han N. Ma Y. Wu Q. Mu Y.. (2021). Enhanced extracellular expression of *α*-amylase Dl3-4-1 in *Bacillus subtilis* via systematic screening of optimal signal peptides. Process Biochem. 108, 176–184. doi: 10.1016/j.procbio.2021.06.018

[ref16] Mishra S. Behera N. (2008). Amylase activity of a starch degrading bacteria isolated from soil receiving kitchen wastes. Afr. J. Biotechnol. 7, 3326–3331. doi: 10.20546/ijcmas.2019.804.071

[ref17] Mukherjee T. Venkata Mohan S. (2021). Metabolic flux of *Bacillus subtilis* under poised potential in electrofermentation system: gene expression vs product formation. Bioresour. Technol. 342:125854. doi: 10.1016/j.biortech.2021.125854, PMID: 34537531

[ref18] Osamura T. Takahashi F. Endo K. Okuda M. Takimura Y. (2023). Autolysis-induced extracellular production of intracellular carboxylesterase EstGtA2 using multiple-protease-deficient *Bacillus subtilis* strains. Biochem. Eng. J. 198:108996. doi: 10.1016/j.bej.2023.108996

[ref19] Rodgman A. Green C. R. (2003). Toxic chemicals in cigarette mainstream smoke - hazard and hoopla. Contributions Tobacco Nicotine Research 20, 481–545. doi: 10.2478/cttr-2013-0764

[ref20] Smets D. Smit J. Xu Y. Karamanou S. Economou A. (2022). Signal peptide-rheostat dynamics delay secretory Preprotein folding. J. Mol. Biol. 434:167790. doi: 10.1016/j.jmb.2022.167790, PMID: 35970402

[ref21] Su Y. Liu C. Fang H. Zhang D. (2020). *Bacillus subtilis*: a universal cell factory for industry, agriculture, biomaterials and medicine. Microb. Cell Factories 19:173. doi: 10.1186/s12934-020-01436-8, PMID: 32883293 PMC7650271

[ref22] Sun J.-G. He J.-W. Wu F.-G. Tu S.-X. Yan T.-J. Si H.. (2011). Comparative analysis on chemical components and sensory quality of aging flue-cured tobacco from four main tobacco areas of China. Agric. Sci. China 10, 1222–1231. doi: 10.1016/S1671-2927(11)60113-2

[ref23] Tian D. Huang L. Zhang Z. Tian Z. Ge S. Wang C.. (2023). A novel approach for quantitative determination of cellulose content in tobacco via 2D Hsqc Nmr spectroscopy. Carbohydr. Res. 526:108790. doi: 10.1016/j.carres.2023.108790, PMID: 36933368

[ref24] Wei X. Zhou Y. Chen J. Cai D. Wang D. Qi G.. (2015). Efficient expression of nattokinase in *Bacillus licheniformis*: host strain construction and signal peptide optimization. J. Ind. Microbiol. Biotechnol. 42, 287–295. doi: 10.1007/s10295-014-1559-4, PMID: 25475755

[ref26] Wong S. L. Ye R. Q. Nathoo S. S. (1994). Engineering and production of streptokinase in a *Bacillus subtilis* expression-secretion system. Appl. Environ. Microbiol. 60, 517–523. doi: 10.1128/aem.60.2.517-523.1994, PMID: 8135514 PMC201342

[ref27] Wu X. C. Lee W. Tran L. Wong S. L. (1991). Engineering a *Bacillus subtilis* expression-secretion system with a strain deficient in six extracellular proteases. J. Bacteriol. 173, 4952–4958. doi: 10.1128/jb.173.16.4952-4958.1991, PMID: 1907264 PMC208183

[ref28] Xing L. Zhang M. Liu L. Hu X. Liu J. Zhou X.. (2023). Multiomics provides insights into the succession of microbiota and metabolite during plant leaf fermentation. Environ. Res. 221:115304. doi: 10.1016/j.envres.2023.115304, PMID: 36649845

[ref29] Yan S. Ren T. Wan Mahari W. A. Feng H. Xu C. Yun F.. (2022). Soil carbon supplementation: improvement of root-surrounding soil bacterial communities, sugar and starch content in tobacco (*N. tabacum*). Sci. Total Environ. 802:149835. doi: 10.1016/j.scitotenv.2021.149835, PMID: 34461468

[ref30] Yan S. Zhao J. Ren T. Liu G. (2020). Correlation between soil microbial communities and tobacco aroma in the presence of different fertilizers. Ind. Crop. Prod. 151:112454. doi: 10.1016/j.indcrop.2020.112454

[ref31] Yang Y. Wang J.-J. Wang C.-X. Li Q. Yang G.-H. (2010). Awareness of tobacco-related health hazards among adults in China. Biomed. Environ. Sci. 23, 437–444. doi: 10.1016/S0895-3988(11)60004-4, PMID: 21315241

[ref32] Yang C. Wu W. Wu S. C. Liu H.-B. (2014). Aroma types of flue-cured tobacco in China: spatial distribution and association with climatic factors. Theor. Appl. Climatol. 115, 541–549. doi: 10.1007/s00704-013-0914-0

[ref33] Yao D. Su L. Li N. Wu J. (2019). Enhanced extracellular expression of *Bacillus stearothermophilus α*-amylase in *Bacillus subtilis* through signal peptide optimization, chaperone overexpression and *α*-amylase mutant selection. Microb. Cell Factories 18:69. doi: 10.1186/s12934-019-1119-8, PMID: 30971250 PMC6458788

[ref34] Ye C. Zhao W. Liu D. Yang R. Cui Z. Zou D.. (2024). Screening, identification, engineering, and characterization of Bacillus-derived *α*-amylase for effective tobacco starch degradation. Int. J. Biol. Macromol. 282:137364. doi: 10.1016/j.ijbiomac.2024.137364, PMID: 39515712

[ref35] Yu J.-F. Chen Q.-L. Ren J. Yang Y.-L. Wang J.-H. Sun X. (2015). Analysis of the multi-copied genes and the impact of the redundant protein coding sequences on gene annotation in prokaryotic genomes. J. Theor. Biol. 376, 8–14. doi: 10.1016/j.jtbi.2015.04.002, PMID: 25865522

[ref36] Zhao Y. Liu X. Qian S. Lu J. Liu X. Cai C.. (2024). Effect of pH and calcium chloride on the thermal inactivation kinetics and stability of chlorophyllase in mulberry leaves. Appl. Food Res. 4:100466. doi: 10.1016/j.afres.2024.100466

