# Supplementary material for: Efficient expression of a novel α-amylase for reduction of tobacco starch and smoke hazard
Source: Front Microbiol. 2025 Jul 9;16:1603337. doi: 10.3389/fmicb.2025.1603337 (PMC12283668; doi:10.3389/fmicb.2025.1603337)

Supplementary Material

1. Supplementary Data

Supplementary Data 1 The gene sequence of five alpha-amylases

The gene sequence of *amyS(WHC-17)*:

ATGTTTAAAAGAATAACAATAGTCGGATTGTCAGTTGTTTTGTTTTTACCTAGTATATAT
GGGAAGAGTAAAGTGTATGCAGATACGATTAACAATGGAACGTTAATGCAGTATTTTGA
GTGGTATGCTCCAAGTGATGGGAATCATTGGAATCGTTTGCGCACTGATGCTGAAAATTT
AGCGCAAAAAGGAATTACATCTGTTTGGATACCTCCTGCATATAAAGGAACTACGCAAA
ATGATGTAGGATATGGAGCATATGATTTATATGATTTGGGTGAATTCAATCAAAAGGGA
ACAGTGCGGACGAAATATGGGACGAAAGCACAATTGAAATCTGCAATTGAAGCTTTACA
TAAGCAAAACATCGATGTATACGGCGATGTAGTTATGAATCATAAAGGTGGAGCAGATT
ATACTGAAACTGTAACAGCTGTTGAGGTAGACCGTAACAATCGAAATGTTGAGGTATCA
GGTGATTATGAAATTAGTGCATGGACAGGGTTTAACTTTCCTGGACGTGGAGATAATTA
TTCTAATTTCAAATGGAAATGGTATCATTTTGACGGAACGGATTGGGATGAAGGAAGGA
AATTAAATCGAATTTATAAATTTAGGGGTATAGGTAAAGCATGGGACTGGGAAGTGTCT
AGTGAGAATGGAAATTATGATTATTTGATGTATGCAGACCTTGATTTTGATCATCCAGAT
GTTGCGAATGAGATGAAAAATTGGGGGACGTGGTATGCGAATGAATTAAATTTAGATGG
CTTTCGTTTAGATGCTGTTAAACATATTGATCATGAATATTTACGCGATTGGGTAAATCA
TGTTAGGCAGCAAACGGGGAAAGAAATGTTTACGGTGGCTGAATATTGGCAAAATGATA
TCCAAACTTTAAATAATTATTTAGCGAAAGTCAATTATAATCAATCTGTATTTGATGCAC
CACTGCATTACAATTTTCATTATGCTTCAAAAGGAAATGGGAATTATGATATGAGAAAT
ATTTTAAATGGAACAGTAATGCAAAATCATCCTGCACTCGCAGTTACTCTTGTTGAGAAT
CATGATTCTCAGCCTGGTCAGTCATTGGAATCTGTAGTAAGTCCGTGGTTTAAACCGTTG
GCATATGCATTTATTTTAACTCGTGCAGAGGGATATCCTTCAGTTTTTTATGGTGATTACT
ATGGGACAAGCGGAAATAGTAGTTATGAAATTCCAGCGTTAAAAGATAAAATTGATCCG
ATTTTGACAGCACGAAAAAACTTTGCATATGGTACGCAGCGTGATTATTTAGACCATCC
AGATGTGATTGGATGGACAAGAGAAGGTGATAGTGTACATGCTAATTCTGGTTTAGCAA
CATTAATCTCTGATGGACCGGGAGGATCAAAGTGGATGGATGTTGGAAAGAATAATGCA
GGGGAAGTATGGCATGATATGACGGGTAATCAAACGAATACTGTAACAATTAATAAGG
ATGGATGGGGGCAATTCCATGTAAGTGGAGGATCAGTTTCCATATATGTTCAGCAGTAA

The gene sequence of *amyE(WHC-84)*:

ATGTTTGCAAAACGATTCAAAACCTCTTTACTGCCGTTATTCGCTGGATTTTTATTGCTGT
TTCATTTGGTTCTGGCAGGACCGGCGGCTGCGAGTGCTGAAACGGCGAACAAATCGAAT
GAGCTTACAGCACCGTCGATCAAAAGCGGAACCATTCTTCATGCATGGAATTGGTCGTT
CAATACGTTAAAACACAATATGAAGGATATTCATGATGCAGGATATACAGCCATTCAGA
CATCTCCGATTAACCAAGTAAAGGAAGGGAATCAAGGAGATAAAAGCATGTCGAACTG
GTACTGGCTGTATCAGCCGACATCGTATCAAATTGGCAACCGTTACTTAGGTACTGAAC
AAGAATTTAAAGAAATGTGTGCAGCCGCTGAAGAATATGGCATAAAGGTCATTGTTGAC
GCGGTCATCAATCATACCACCAGTGATTATGCCGCGATTTCCAATGAGGTTAAGAGTATT
CCAAACTGGACACATGGAAACACACAAATTAAAAACTGGTCTGATCGATGGGATGTCAC
GCAGAATTCATTGCTCGGGCTGTATGACTGGAATACACAAAATACACAAGTACAGTCCT

ATCTGAAACGGTTCTTAGACAGGGCATTGAATGACGGGGCAGACGGTTTTCGATTTGAT
GCCGCCAAACATATAGAGCTTCCAGATGATGGCAGTTACGGCAGTCAATTTTGGCCGAA
TATCACAAATACATCTGCAGAGTTCCAATACGGAGAAATCCTGCAGGATAGTGCCTCCA
GAGATGCTGCATATGCGAATTATATGGATGTGACAGCGTCTAACTATGGGCATTCCATA
AGGTCCGCTTTAAAGAATCGTAATCTGGGCGTGTCGAATATCTCCCACTATGCATCTGAT
GTGTCTGCGGACAAGCTAGTGACATGGGTAGAGTCGCATGATACGTATGCCAATGATGA
TGAAGAGTCGACATGGATGAGCGATGATGATATCCGTTTAGGCTGGGCGGTGATAGCTT
CTCGTTCAGGCAGTACGCCTCTTTTCTTTTCCAGACCTGAGGGAGGCGGAAATGGTGTGA
GGTTCCCGGGGAAAAGCCAAATAGGCGATCGCGGGAGTGCTTTATTTGAAGATCAGGCT
ATCACTGCGGTCAATAGATTTCACAATGTGATGGCTGGACAGCCTGAGGAACTCTCGAA
CCCGAATGGAAACAACCAGATATTTATGAATCAGCGCGGCTCACATGGCGTTGTGCTGG
CAAATGCAGGTTCATCCTCTGTCTCTATCAATACGGCAACAAAATTGCCTGATGGCAGGT
ATGACAATAAAGCTGGAGCGGGTTCATTTCAAGTGAACGATGGTAAACTGACAGGCACG
ATCAATGCCAGGTCTGTAGCTGTGCTTTATCCTGATGATATTGCAAAAGCGCCTCATGTT
TTCCTTGAGAATTACAAAACAGGTGTAACACATTCTTTCAATGATCAACTGACGATTACC
TTGCGTGCAGATGCGAATACAACAAAAGCCGTTTATCAAATCAATAATGGACCAGAGAC
GGCGTTTAAGGATGGAGATCAATTCACAATCGGAAAAGGAGATCCATTTGGCAAAACAT
ACACCATCATGTTAAAAGGAACGAACAGTGATGGTGTAACGAGGACCGAGAAATACAG
TTTTGTTAAAAGAGATCCAGCGTCGGCCAAAACCATCGGCTATCAAAATCCGAATCATT
GGAGCCAGGTAAATGCTTATATCTATAAACATGATGGGAGCCGAGTAATTGAATTGACC
GGATCTTGGCCTGGAAAACCAATGACTAAAAATGCAGACGGAATTTACACGCTGACGCT
GCCTGCGGACACGGATACAACCAACGCAAAAGTGATTTTTAATAATGGCAGCGCCCAAG
TGCCCGGTCAGAATCAGCCTGGCTTTGATTACGTGCTAAATGGTTTATATAATGACTCGG
GCTTAAGCGGTTCTCTTCCCCATTGA

The gene sequence of *amyE(WHC-115)*:

ATGTTTGCAAAACGATTCAAAACCTCTTTACTGCCGTTATTCGCTGGATTTTTATTGCTGT
TTCATTTGGTTCTGGCAGGACCGGCGGCTGCGAGTGCTGAAACGGCGAACAAATCGAAT
GAGCTTACAGCACCGTCGATCAAAAGCGGAACCATTCTTCATGCATGGAATTGGTCGTT
CAATACGTTAAAACACAATATGAAGGATATTCATGATGCAGGATATACAGCCATTCAGA
CATCTCCGATTAACCAAGTAAAGGAAGGGAACCAAGGAAATAAAAGCATGTCGAACTG
GTACTGGCTCTATCAGCCGACATCGTACCAAATTGGCAACCGTTACTTAGGTACTGAAC
AAGAATTTAAAGAAATGTGTGCAGCCGCTGAAGAATATGGCATAAAGGTCATTGTTGAC
GCGGTCATCAATCATACCACCAGTGACTATGCCGCGATTTCCAATGAAATTAAGAGTAT
TCCAAACTGGACACATGGAAACACACAAATTAAAAACTGGTCTGATCGATGGGATGTCA
CGCAGAATTCATTGCTCGGGCTGTATGACTGGAATACACAAAATACACAAGTACAGTCC
TATTTGAAACGGTTCTTAGAAAGAGCGTTGAATGACGGGGCAGACGGTTTTCGCTTTGA
TGCCGCCAAACATATAGAGCTTCCGGATGATGGGAGTTACGGCAGCCAATTTTGGCCGA
ATATCACAAACACATCTGCAGAGTTTCAATACGGAGAAATCCTGCAGGATAGTGCTTCC
AGAGATGCTTCATATGCGAATTATATGAATGTGACAGCGTCTAACTATGGGCATTCCAT
AAGGTCCGCTTTAAAGAATCGCAATCTGGGCGTGTCGAATATCTCCCACTATGCATCTGA
TGTGTCTGCGGACAAGCTAGTGACATGGGTGGAGTCGCATGATACGTATGCCAATGATG
ATGAAGAGTCGACATGGATGAGCGATGATGATATCCGTTTAGGCTGGGCGGTGATAGCT
TCTCGTTCAGGCAGTACGCCTCTTTTCTTTTCCAGACCTGAGGGAGGCGGAAATGGTGTG
AGGTTCCCGGGGAAAAGCCAAATAGGCGATCGCGGGAGTGCTTTATTTGAAGATCAGGC
TATCACTGCGGTCAATAGATTTCACAATGTGATGGCTGGACAGCCTGAGGAACTCTCGA

ACCCGAATGGAAACAACCAGATATTTATGAATCAGCGCGGCTCACATGGCGTTGTGCTG
GCAAATGCAGGTTCATCCTCTGTCTCTATCAATACGGCAACAAAATTGCCTGATGGCAG
GTATGACAATAAAGCTGGAGCGGGTTCATTTCAAGTGAACGATGGTAAACTGACAGGCA
CGATCAATGCCAGGTCTGTAGCTGTGCTTTATCCTGATGATATTGCAAAAGCGCCTCATG
TTTTCCTTGAGAATTACAAAACAGGTGTAACACATTCTTTCAATGATCAACTGACGATTA
CCTTGCGTGCAGATGCGAATACAACAAAAGCCGTTTATCAAATCAATAATGGACCAGAG
ACGGCGTTTAAGGATGGAGATCAATTCACAATCGGAAAAGGAGATCCATTTGGCAAAAC
ATACACCATCATGTTAAAAGGAACGAACAGTGATGGTGTAACGAGGACCGAGAAATAC
AGTTTTGTTAAAAGAGATCCAGCGTCGGCCAAAACCATCGGCTATCAAAATCCGAATCA
TTGGAGCCAGGTAAATGCTTATATCTATAAACATGATGGGAGCCGAGTAATTGAATTGA
CCGGATCTTGGCCTGGAAAACCAATGACTAAAAATGCAGACGGAATTTACACGCTGACG
CTGCCTGCGGACACGGATACAACCAACGCAAAAGTGATTTTTAATAATGGCAGCGCCCA
AGTGCCCGGTCAGAATCAGCCTGGCTTTGATTACGTGCTAAATGGTTTATATAATGACTC
GGGCTTAAGCGGTTCTCTTCCCCATTGA

The gene sequence of *amyE(WHC-117)*:

ATGTTTGCAAAACGATTCAAAACCTCTTTACTGCCGTTATTCGCTGGATTTTTATTGCTGT
TTCATTTGGTTCTGGCAGGACCGGCGGCTGCGAGTGCTGAAACGGCGAACAAATCGAAT
GAGCTTACAGCACCGTCGATCAAAAGCGGAACCATTCTTCATGCATGGAATTGGTCGTT
CAATACGTTAAAACATAATATGAAGGATATTCATGATGCAGGATATACAGCCATTCAGA
CATCTCCGATTAACCAAGTAAAGGAAGGGAACCAAGGAAATAAAAGCATGTCGAACTG
GTACTGGCTCTATCAGCCGACATCGTACCAAATTGGCAACCGTTACTTAGGAACTGAAC
AAGAATTTAAAGAAATGTGTGCAGCCGCTGAAGAATATGGCATAAAGGTCATTGTTGAC
GCGGTCATCAATCATACCACCAGTGATTATGCCGCGATTTCCAATGAGATTAAGAGTATT
CCAAACTGGACACATGGAAACACGCAAATTAAAAACTGGTCTGATCGATGGGATGTCAC
GCAGAATTCATTGCTCGGGCTGTATGACTGGAATACACAAAATACACAAGTACAGTCCT
ATTTGAAACGGTTCTTAGAAAGAGCATTGAATGACGGGGCAGATGGATTTCGCTTTGAT
GCCGCTAAACATATAGAGCTTCCGGATGATGGGAGTTACGGCAGTCAATTTTGGCCGAA
TATCACAAATACATCTGCAGAGTTCCAATACGGAGAAATCCTGCAGGATAGTGCCTCCA
GAGATGCTGCATATGCGAATTATATGAATGTGACAGCGTCTAACTATGGGCATTCCATA
AGGTCCGCTTTAAAGAATCGTAATCTGGGCGTGTCGAATATCTCCCACTATGCATCTGAT
GTGTCAGCGGACAAGCTAGTGACATGGGTAGAGTCGCATGATACGTATGCCAATGATGA
TGAAGAGTCGACATGGATGAGCGATGATGATATCCGTTTAGGCTGGGCGGTGATAGCTT
CTCGTTCAGGCAGTACGCCTCTTTTCTTTTCCAGACCTGAGGGAGGCGGAAATGGTGTGA
GATTCCCGGGGAAAAGCCAAATAGGCGATCGCGGGAGTGCTTTATTTGAAGATCAGGCT
ATCACTGCGGTCAATAGATTTCACAATGTGATGGCTGGACAGCCTGAGGAGCTCTCGAA
TCCGAATGGAAACAACCAGATATTTATGAATCAGCGCGGCTCACATGGCGTTGTGCTGG
CAAATGCAGGTTCATCCTCTGTTTCTATCAATACGCCAACAAAATTGCCTGATGGCAGGT
ATGACAATAAAGCTGGGGCAGGTTCATTTCAAGTGAATGATGGTAAACTGACAGGCACG
ATCAATGCCAGGTCTGTGGCTGTGCTTTATCCTGATGATATTGCAAAAGCGCCTCATGTT
TTTCTTGAGAATTACAAAACAGGTGTAACACATTCTTTCAATGATCAACTGACGATTACC
TTGCGTGCAGATGCGAATACAACAAAAGCCGTTTATCAAATCAATAATGGACCAGAGAC
GGCGTTTAAGGATGGAGATCAATTCACAATCGGAAAAGGAGATCCATTTGGCAAAACAT
ACACCATCATGTTAAAAGGAACGAACAGTGATGGTGTAACGAAGGCCGAGGAATACAG
TTTTGTTAAAAGAGATCCAGCTTCGGCCAAAACCATCGGCTATCAAAATCCGAATCATT
GGAGCCAGGTAAATGCTTATATCTATAAACATGATGGGAGCCGGGCAATTGAATTGACC
GGATCTTGGCCTGGAAAACCAATGACTAAAAATGCAGACGGAATTTACACGCTGACTCT

GCCTGCGGACACGGATACAACCAACGCAAAAGTGATTTTTAATAATGGCAGCGCCCAAG
TGCCCGGCCAGAATCAGCCTGGCTTTGATTACGTGCAAAATGGTTTATATAATGACTCGG
GCTTAAGCGGTTCTCTTCCCCATTGA

The gene sequence of *amyA(LC)* :

ATGATTCAAAAACGAAAGCGGACAGTTTCGTTCAGACTTGTGCTTATGTGCACGCTGTTA
TTTGTCAGTTTGCCGATTACAAAAACATCAGCCGTAAATGGCACGCTGATGCAGTATTTT
GAATGGTATACGCCGAACGACGGCCAGCATTGGAAACGATTGCAGAATGATGCGGAAC
ATTTATCGGATATCGGAATCACTGCCGTCTGGATTCCTCCCGCATACAAAGGATTGAGCC
AATCCGATAACGGATACGGACCTTATGATTTGTATGATTTAGGAGAATTCCAGCAAAAA
GGGACGGTCAGAACGAAATACGGCACAAAATCAGAGCTTCAAGATGCGATCGGCTCAC
TGCATTCCCGGAACGTCCAAGTATACGGAGATGTGGTTTTGAATCATAAGGCTGGTGCT
GATGCAACAGAAGATGTAACTGCCGTCGAAGTCAATCCGGCCAATAGAAATCAGGAAA
CTTCGGAGGAATATCAAATCAAAGCGTGGACGGATTTTCGTTTTCCGGGCCGTGGAAAC
ACGTACAGTGATTTTAAATGGCATTGGTATCATTTCGACGGAGCGGACTGGGATGAATC
CCGGAAGATCAGCCGCATCTTTAAGTTTCGTGGGGAAGGAAAAGCGTGGGATTGGGAA
GTATCAAGTGAAAACGGCAACTATGACTATTTAATGTATGCTGATGTTGACTACGACCA
CCCTGATGTCGTGGCAGAGACAAAAAAATGGGGTATCTGGTATGCGAATGAACTGTCAT
TAGACGGCTTCCGTATTGATGCCGCCAAACATATTAAATTTTCATTTCTGCGTGATTGGG
TTCAGGCGGTCAGACAGGCGACGGGAAAAGAAATGTTTACGGTTGCGGAGTATTGGCA
GAATAATGCCGGGAAACTCGAAAACTACTTGAATAAAACAAGCTTTAATCAATCCGTGT
TTGATGTTCCGCTTCATTTCAATTTACAGGCGGCTTCCTCACAAGGAGGCGGATATGATA
TGAGGCGTTTGCTGGACGGTACCGTTGTGTCCAGGCATCCGGAAAAGGCGGTTACATTT
GTTGAAAATCATGACACACAGCCGGGACAGTCATTGGAATCGACAGTCCAAACTTGGTT
TAAACCGCTTGCATACGCCTTTATTTTGACAAGAGAATCCGGTTATCCTCAGGTGTTCTA
TGGGGATATGTACGGGACAAAAGGGACATCGCCAAAGGAAATTCCCTCACTGAAAGAT
AATATAGAGCCGATTTTAAAAGCGCGTAAGGAGTACGCATACGGGCCCCAGCACGATTA
TATTGACCACCCGGATGTGATCGGATGGACGAGGGAAGGTGACAGCTCCGCCGCCAAAT
CAGGTTTGGCCGCTTTAATCACGGACGGACCCGGCGGATCAAAGCGGATGTATGCCGGC
CTGAAAAATGCCGGCGAGACATGGTATGACATAACGGGCAACCGTTCAGATACTGTAAA
AATCGGATCTGACGGCTGGGGAGAGTTTCATGTAAACGATGGGTCCGTCTCCATTTATGT
TCAGAAATAA

2. Supplementary Figures and Tables

Supplementary Table 1 Primers used in this study.

Primer	Sequence of primer (5' to 3')
P_{43}-F	GC**TCTAGA**TGATAGGTGGTATGTTTTCGC
P_{43}-R	CGC**GGATCC**TTCATGTGTACATTCCTCTCTTAC
P_{srfA}-F	GC**TCTAGA**GACAAAAATGTCATGAAAGAATCG
P_{srfA}-R	CGC**GGATCC**GGGTAAAAAGTTATTTCCATATTGTC
27F	AGAGTTTGATCCTGGCTCAG
1492R	GGTTACCTTGTTACGACTT
pHY300-F	GTTTATTATCCATACCCTTAC
pHY300-R	CAGATTTCGTGATGCTTGTC
amyA-F	GC**TCTAGA**ATGTTTGAAAAACGATTCAAAAC
amyA-R	CGC**GGATCC**TTAATGCGGAAGATAACCGT
amyE-F	GC**TCTAGA**ATGTTTGCAAAACGATTCAAAACC
amyE-R	CGC**GGATCC**TCAATGGGGAAGAGAACCG
AmyS-F	GC**TCTAGA**ATGTTTAAAAGAATAACAATAGTCGGATT
AmyS-R	CGC**GGATCC**TTACTGCTGAACATATATGGAAACTGAT
amy(L)-F	CGC**GGATCC**ATGAGAGGGAGAGGAAACATG
amy(L)-R	AATCCGTCCTCTCTGCTCTTTTATTTCTGAACATAAATGGAGACGGA
Tamyl-F	TCCGTCTCCATTTATGTTCAGAAATAAAAGAGCAGAGAGGACGGATT
Tamyl-R	TCC**CCCGGG**CGCAATAATGCCGTCGCACT
Singnal1-F	GC**TCTAGA**GTGAAAGAAGTAAGGTTTTGGG
Singnal1-R	GCATCAGCGTGCCATTTACAGCCTTACTAACTAACGGTATCAC
amy(LS-1)-F	GTGATACCGTTAGTTAGTAAGGCTGTAAATGGCACGCTGATGC
amy(LS-1)-R	TCC**CCCGGG**TTATTTCTGAACATAAATGGAGAC
Singnal2-F	GC**TCTAGA**ATGCCTTATCTGAAACGAGTGT
Singnal2-R	GCATCAGCGTGCCATTTACAGCTGAGGCAGTAGCAGTGACTGCAAAC
amy(LS-2)-F	GTTTGCAGTCACTGCTACTGCCTCAGCTGTAAATGGCACGCTGAT

	GC
amy(LS-2)-R	TCC**CCCGGG**TTATTTCTGAACATAAATGGAGAC
Singnal3-F	GC**TCTAGA**ATGCGCATTTTCAAAAAAG
Singnal3-R	GCATCAGCGTGCCATTTACAGCATGTGCTGTATTCACATTTAC
amy(LS-3)-F	GTAAATGTGAATACAGCACATGCTGTAAATGGCACGCTGATGC
amy(LS-3)-R	TCC**CCCGGG**TTATTTCTGAACATAAATGGAGAC

Note: Restriction sites highlight in bold. Underline stands for the overlap region for Splicing by Overlapping Extension PCR (SOE-PCR).

Supplementary Figure 1 The α-amylase gene fragments amplified from different strains A: *amyS(WHC-17)* (Lane 1)； B: *amyE(WHC-84)* (Lane 2); C: *amyE(WHC-115)* fragmen； D: *amyE(WHC-117)* (Lane 1)；E: *amyA(LC)* (Lane 1).

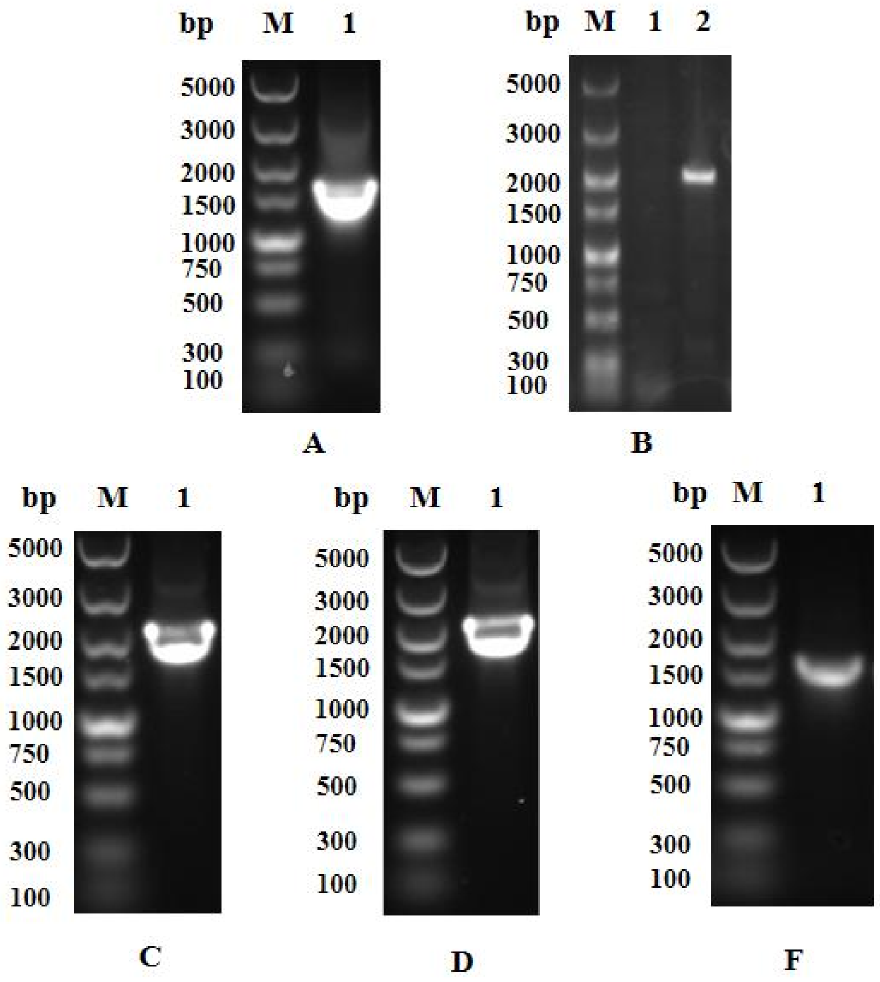

Supplementary Figure 2 Multiple sequence alignment of amino acid sequences of different α-amylases

Sequence type explicitly set to Protein

Sequence format is Pearson

Sequence 1: A *amyS(WHC-17)* **513 aa**

Sequence 2: B *amyE(WHC-84)* **659 aa**

Sequence 3: C *amyE(WHC-115)* **659 aa**

Sequence 4: D *amyE(WHC-117)* **659 aa**

Sequence 5: E *amyA(LC)* **514 aa**

Sequences (1:2) Aligned. Score: 10.9162

Sequences (1:3) Aligned. Score: 11.306

Sequences (1:4) Aligned. Score: 11.306

Sequences (1:5) Aligned. Score: 67.4464

Sequences (2:3) Aligned. Score: 99.2413

Sequences (2:4) Aligned. Score: 98.4825

Sequences (2:5) Aligned. Score: 12.0623

Sequences (3:4) Aligned. Score: 98.9378

Sequences (3:5) Aligned. Score: 11.284

Sequences (4:5) Aligned. Score: 11.4786

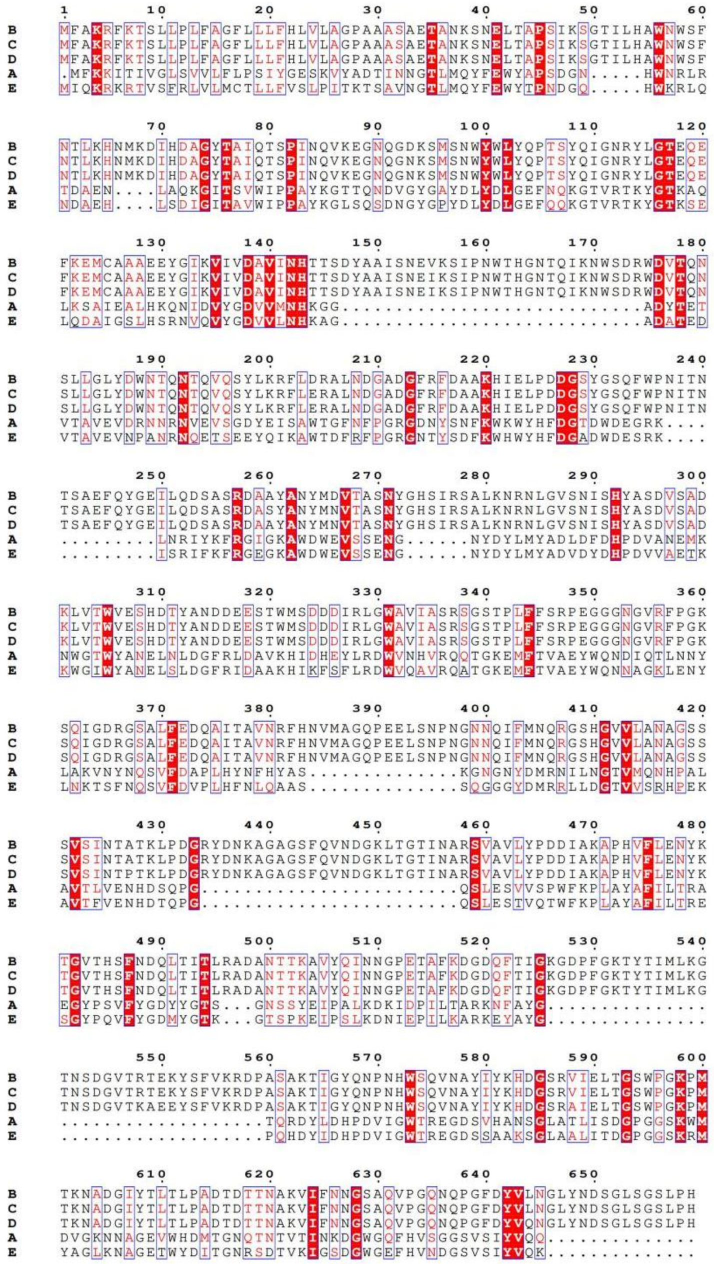

Supplementary Figure 3 Initial Signal peptide information of α-amylase amy(LC)

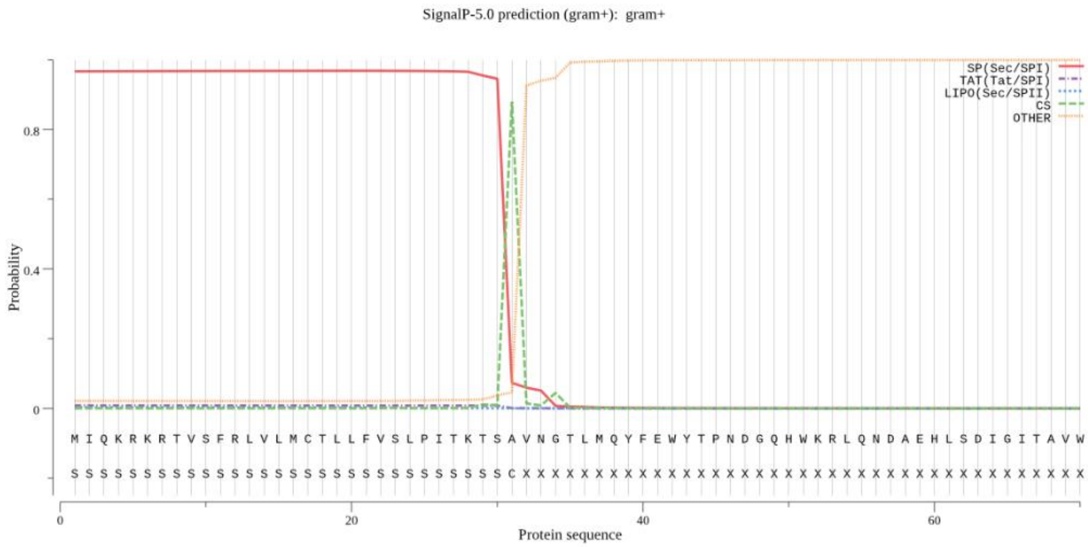

Supplementary Figure 4 The α-amylase gene fragments amplified from different strains (Full scan of the entire original gel, uncropped and unedited version, This image is intended to suppor Supplementaryt Figure 1): A: *amyS(WHC-17)* ； B: *amyE(WHC-84)*; C: *amyE(WHC-115)* fragmen； D: *amyE(WHC-117)*； E: *amyA(LC)*.

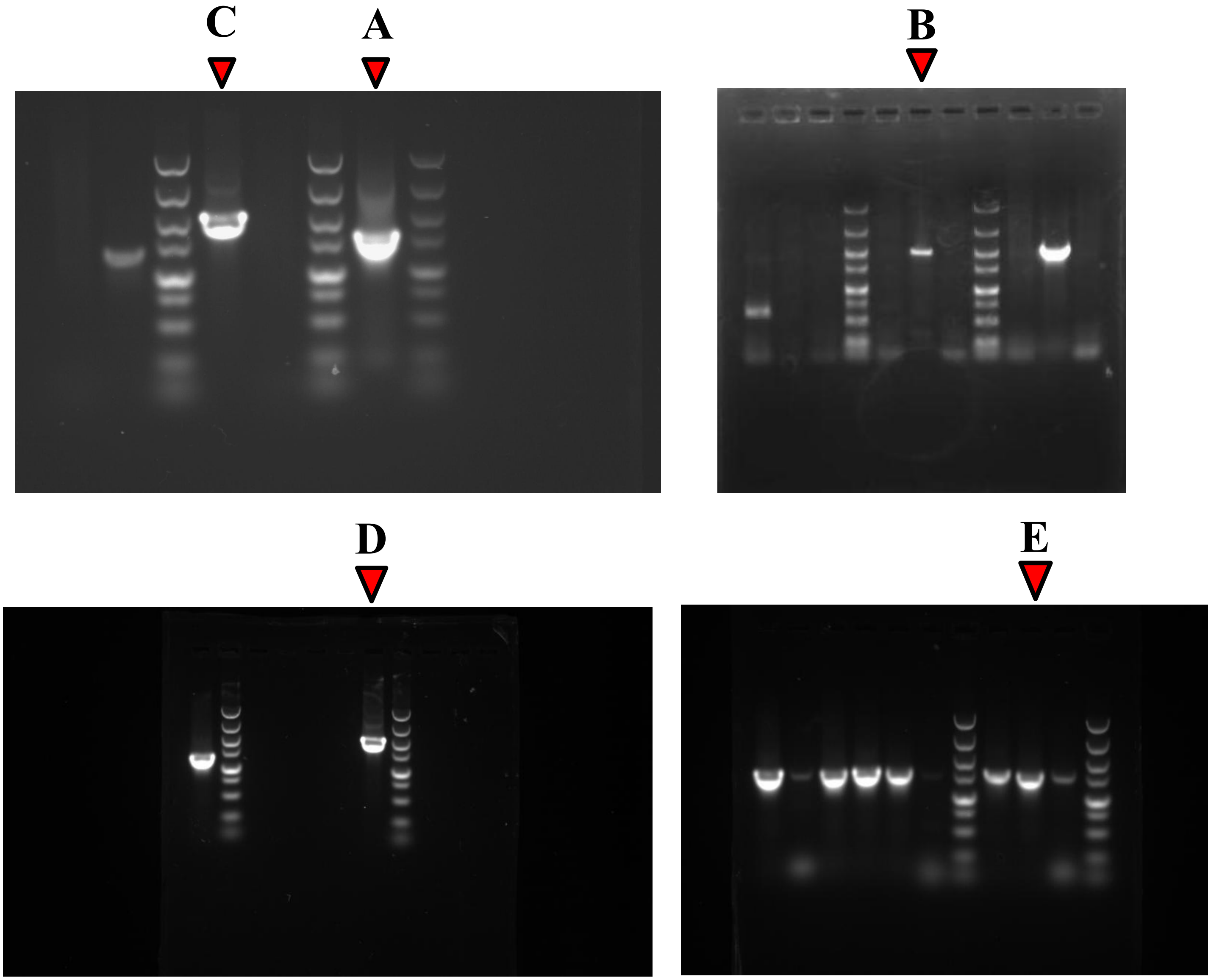

Supplementary Figure 5 Full scan of the entire original gel of the SDS-PAGE result in Figure 5C in the manuscript (uncropped and unedited version)

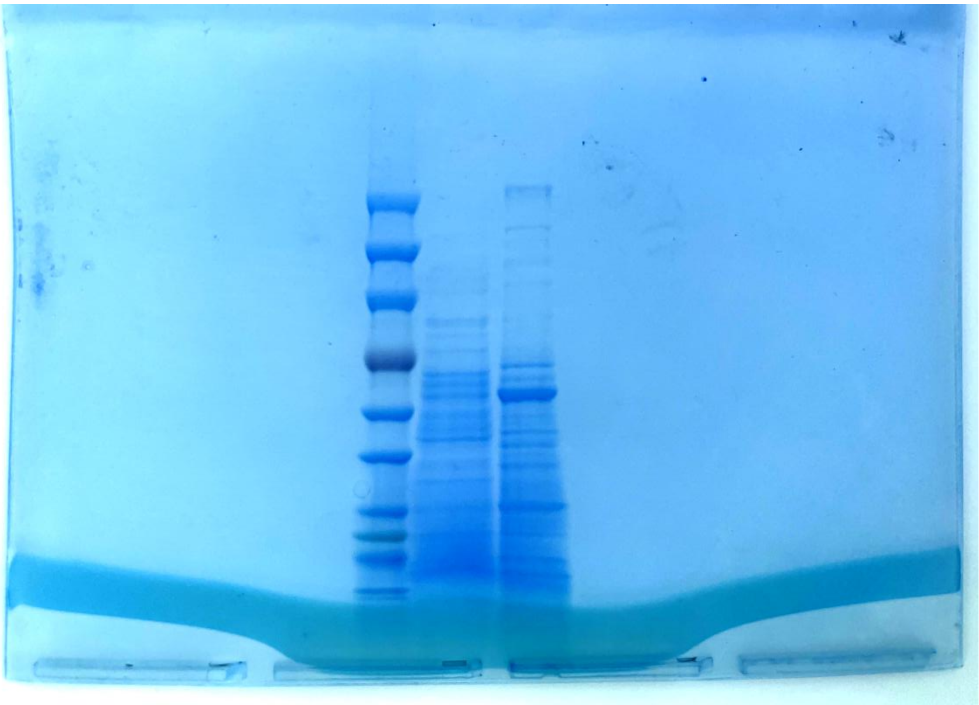

Supplement: Supplementary file 1 [file Data_Sheet_1.pdf]
